# Serum/plasma biomarkers and the progression of cardiometabolic multimorbidity: a systematic review and meta-analysis

**DOI:** 10.3389/fpubh.2023.1280185

**Published:** 2023-11-23

**Authors:** Yichen Jin, Ziyuan Xu, Yuting Zhang, Yue Zhang, Danyang Wang, Yangyang Cheng, Yaguan Zhou, Muhammad Fawad, Xiaolin Xu

**Affiliations:** ^1^School of Public Health, The Second Affiliated Hospital, Zhejiang University School of Medicine, Hangzhou, Zhejiang, China; ^2^The Key Laboratory of Intelligent Preventive Medicine of Zhejiang Province, Hangzhou, Zhejiang, China; ^3^School of Public Health, Faculty of Medicine, The University of Queensland, Brisbane, QLD, Australia

**Keywords:** biomarker, cardiometabolic multimorbidity, type 2 diabetes mellitus, coronary heart disease, stroke, systematic review

## Abstract

**Background:**

The role of certain biomarkers in the development of single cardiometabolic disease (CMD) has been intensively investigated. Less is known about the association of biomarkers with multiple CMDs (cardiometabolic multimorbidity, CMM), which is essential for the exploration of molecular targets for the prevention and treatment of CMM. We aimed to systematically synthesize the current evidence on CMM-related biomarkers.

**Methods:**

We searched PubMed, Embase, Web of Science, and Ebsco for relevant studies from inception until August 31st, 2022. Studies reported the association of serum/plasma biomarkers with CMM, and relevant effect sizes were included. The outcomes were five progression patterns of CMM: (1) no CMD to CMM; (2) type 2 diabetes mellitus (T2DM) followed by stroke; (3) T2DM followed by coronary heart disease (CHD); (4) T2DM followed by stroke or CHD; and (5) CHD followed by T2DM. Newcastle-Ottawa Quality Assessment Scale (NOS) was used to assess the quality of the included studies. A meta-analysis was conducted to quantify the association of biomarkers and CMM.

**Results:**

A total of 68 biomarkers were identified from 42 studies, which could be categorized into five groups: lipid metabolism, glycometabolism, liver function, immunity, and others. Lipid metabolism biomarkers were most reported to associate with CMM, including TC, TGs, HDL-C, LDL-C, and Lp(a). Fasting plasma glucose was also reported by several studies, and it was particularly associated with coexisting T2DM with vascular diseases. According to the quantitative meta-analysis, HDL-C was negatively associated with CHD risk among patients with T2DM (pooled OR for per 1 mmol/L increase = 0.79, 95% CI = 0.77–0.82), whereas a higher TGs level (pooled OR for higher than 150 mg/dL = 1.39, 95% CI = 1.10–1.75) was positively associated with CHD risk among female patients with T2DM.

**Conclusion:**

Certain serum/plasma biomarkers were associated with the progression of CMM, in particular for those related to lipid metabolism, but heterogeneity and inconsistent findings still existed among included studies. There is a need for future research to explore more relevant biomarkers associated with the occurrence and progression of CMM, targeted at which is important for the early identification and prevention of CMM.

## 1 Introduction

With the growth of the aging population, non-communicable diseases (NCDs) have become the major global disease burden and the leading cause of death worldwide (1). Some NCDs may share similar pathogenesis or identical risk factors (2, 3), leading to their simultaneous coexistence in individuals and resulting in multimorbidity (i.e., the coexistence of two or more NCDs) (4). Cardiometabolic multimorbidity (CMM) is one of the most studied patterns of multimorbidity (5), defined as the co-occurrence of two or more cardiometabolic diseases (CMDs), including coronary heart disease (CHD), stroke, and type 2 diabetes mellitus (T2DM) (2, 6, 7). The prevalence of CMM has increased rapidly in the past few decades (8), potentially resulting in worse quality of life, excess morbidity, and mortality (8–11).

Current studies on the risk factors of CMM mainly focused on some macro or external factors, such as lifestyle factors (12), dietary factors (6, 13), and environmental factors (14). Limited studies have explored the role of micro risk factors, such as serum/plasma biomarkers, on CMM. Biomarkers are the most objective and quantifiable medical markers that can be measured and are commonly used as clinical and diagnostic tools (15). Changes in biomarker levels can reflect the interaction effects among genetic factors, lifestyle factors, environmental factors, and health conditions, which can be used to explore new routes of disease occurrence, improve the accuracy of risk prediction, and achieve stratifying prevention and management of these diseases (16, 17). The relationship between specific biomarkers and single CMD was extensively investigated. For example, high levels of triglycerides (TGs), low-density lipoprotein cholesterol (LDL-C) and fasting plasma glucose (FPG), low levels of high-density lipoprotein cholesterol (HDL-C) have been reported to be risk factors of cardiovascular complications in T2DM patients (18–20). A 12-year follow-up longitudinal analysis revealed that high homocysteine (tHcy) and low Methionine (Met) levels were associated with a higher risk of cardiovascular multimorbidity in older age (21). However, there remains a lack of studies investigating the relationship between biomarkers and CMM. Moreover, no systematic review and meta-analysis has yet synthesized existing evidence on the association between biomarkers and the progression of CMM, which is critical for the clinical practice of identifying high-risk populations early via laboratory blood testing. We aimed to synthesize the available scattered evidence on the role of serum/plasma biomarkers in the development and progression of CMM.

## 2 Methods

### 2.1 Search strategy

This systematic review was conducted according to the Preferred Reporting Items for Systematic Reviews and Meta-Analyses (PRISMA) guidelines (22). Databases, including PubMed, Embase, Web of Science, and Ebsco, were searched for eligible articles published in English from January 1^st^, 1900, to August 31^st^, 2022. Details of search strategies are presented in Supplementary Table S1. Reference lists and Google were also searched to identify any additional or gray literature that met the inclusion criteria.

### 2.2 Selection criteria

After removing the duplicate records, three authors (JY, XZ, ZY) independently screened a third of the total records for titles and abstracts and evaluated the full text to select eligible articles with exclusion reasons recorded. A total of 5% of the records were randomly selected and cross-checked for verification. Inter-examiner agreements across the three authors were calculated using Cohen κ statistics, with ranges of 0.01–0.20 representing slight agreement, 0.21–0.40 representing fair agreement, 0.41–0.60 representing moderate agreement, 0.61–0.80 representing substantial agreement, and 0.81–0.99 representing almost perfect agreement (23). Discrepancies were discussed with a fourth author (XX) in regular group meetings.

Inclusion criteria were: (1) observational studies such as cohort studies and nested case-control studies; (2) the primary outcome of the study was CMM, which was defined as the co-occurrence of at least two of the following CMDs: CHD, T2DM, and stroke. Myocardial infarction (MI), ischemic heart disease (IHD), angina pectoris, coronary artery bypass grafting, percutaneous transluminal coronary angioplasty, coronary revascularization procedures, and CHD-related death are all examples of CHD occurrences; (3) biomarkers include all detectable and quantifiable biochemical parameters found in plasma or serum, except for gene regulatory molecules such as microRNA. Laboratory examinations were conducted at baseline or before the occurrence of CMM; (4) participants were free of CMM at baseline; (5) effect sizes [e.g., hazard ratio (HR), odds ratio (OR), relative risk (RR), and 95% confidence interval (CI)] of the biomarkers on CMM were reported; and (6) written in English.

### 2.3 Quality assessment and data extraction

Three authors (JY, XZ, ZY) independently extracted data from all included articles using a pre-designed standardized data extraction form, including title, first author, year of publication, country, sex distribution and age of participants, sample size, follow-up duration, biomarkers, definition of CMM, number (percentage) of participants developing CMM, comparison, adjusted effect sizes of biomarkers on CMM, and covariates. Disagreements on the extraction were discussed with a fourth author (XX).

Three authors independently (JY, XZ, ZY) assessed the methodological quality of each study using Newcastle-Ottawa Quality Assessment Scale (NOS) (24), with discrepancies discussed with a fourth author (XX). Studies scored ≥7 (out of 9) were considered as high quality, and those scored ≤ 3 were of low quality (25).

### 2.4 Evidence synthesis

A narrative synthesis approach was used to summarize the effect sizes of specific biomarkers on different progression patterns of CMM, which was visualized through a heatmap. However, due to the heterogeneity in the cut-off points or measurement units of biomarkers among included articles, we could not pool the effect sizes of the majority of biomarkers on CMM through meta-analysis. For eligible biomarkers with enough data for meta-analysis, different measurement levels of specific biomarkers were converted into a consistent standard. Categorical variables were converted into binary forms, while effect sizes of continuous variables were transformed into ORs according to the following formulas (26):


(1) $$\begin{array}{l} \text{O}{\text{R}}_{\;\left(\text{standardized}\right)}=OR_{\;\left(original\right)}^{\;Increment\;\left(standardized\right)/Increment\;\left(original\right)} \end{array}$$

The heterogeneity was assessed using Cochran's *Q*-statistic and *I*^2^ statistics, with thresholds of 25%, 50%, and 75% for low, moderate, and high heterogeneity (23). Depending on the degree of heterogeneity, either a fixed effect model or a random effect model was used to estimate the pooled ORs (95% CIs). We weighted studies using the inverse-variance approach. The meta-analysis was conducted using Review Manager (RevMan) Version 5.4. (Copenhagen, Denmark: The Nordic Cochrane Center, The Cochrane Collaboration).

## 3 Results

### 3.1 Characteristics of included studies

**Figure 1:**
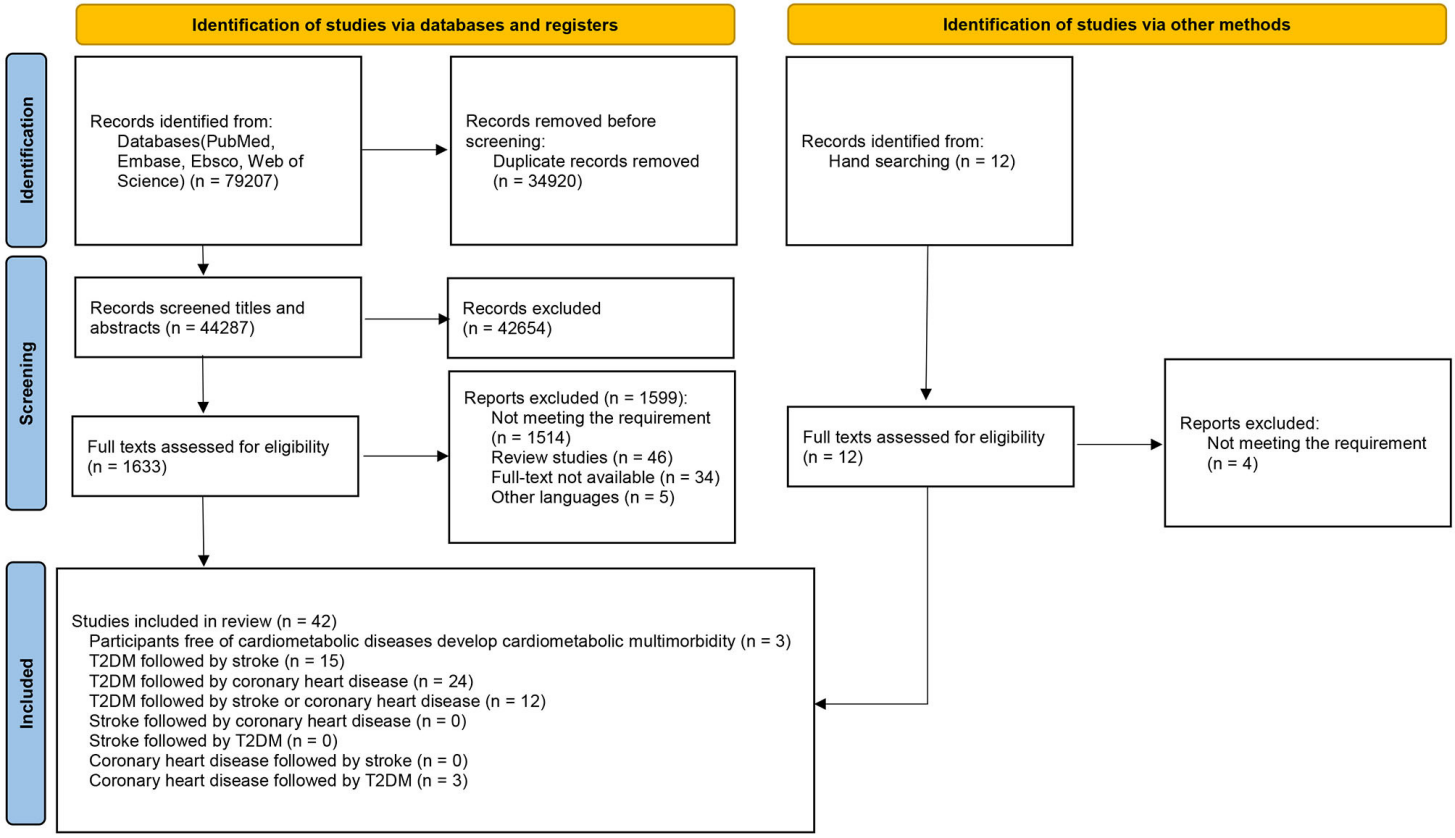
Flowchart of study selection according to the PRISMA guideline.

A total of 79,207 publications were initially identified through a database search. After screening for titles and abstracts, 1,633 were selected for full-text review (inter-reader agreement κ = 0.81/0.85/0.83). A total of 42 studies (7, 27–67) that met the inclusion criteria were finally included in our review after the full-text screen (inter-reader agreement κ=0.86/0.88/0.82). The selection process is shown in Figure 1, and the basic characteristics of included studies are presented in Supplementary Table S2. Among the 42 studies included in our review, we selected five of them for the meta-analysis (40, 43, 48, 49, 62) (Table 1), which were prospective studies focusing on the associations of FPG, LDL-C, HDL-C, and TGs with the progression of CMM among T2DM patients based on populations from Japan, Greece, and Italy. These 42 studies were grouped into five categories by different progression patterns of CMM: (1) participants were free of CMD at baseline and developed CMM during follow-up (*n* = 3); (2) T2DM followed by stroke (*n* = 15); (3) T2DM followed by CHD (*n* = 24); (4) T2DM followed by stroke or CHD (*n* = 12); and (5) CHD followed by T2DM (*n* = 3).

**Table 1:** Characteristics of the studies included in the meta-analysis.

**Study**	**Country**	**Mean age**	***n* (female, %)**	**Follow-up years**	**Definition of CMM**	**No. of events**	**Comparison**	**HR (95% CI)**	**Covariates**
**FPG**									
Hayashi et al. (48)	Japan	67.4	4,014 (48.2)	5.5	Patients with T2DM developed IHD (Definite fatal and nonfatal MI or angina pectoris);	153	Per 10 mg/dL higher	1.00 (0.99–1.01);	NA
					Ischemic stroke or primary intracerebral hemorrhage	104		1.01 (0.99–1.01)	
Sone et al. (62)	Japan	58.2	1,771 (46.9)	7.86 (median)	Patients with T2DM developed CHD (angina pectoris and MI);	109	Per 1 mmol/L higher	0.99 (0.91–1.09);	Gender, age, diabetes duration, body mass index, systolic blood pressure, HbA1c, LDL cholesterol, HDL cholesterol, triglycerides, smoking status, and alcohol intake
					Stroke	85		1.02 (0.91–1.13)	
**LDL-C**									
Protopsaltis et al. (49)	Greece	60.4	599 (46.0)	10.1 (median)	Patients with T2DM developed ischemic stroke	78	Per 1 mg/dL	1.01 (0.99–1.02)	Gender, age, smoking, body mass index, HbA1C, lipids, and diabetes duration
Sone et al. (62)	Japan	58.2	1,771 (46.9)	7.86 (median)	Patients with T2DM developed stroke	85	Per 1 mmol/L higher	1.00 (0.76–1.32)	Gender, age, diabetes duration, body mass index, systolic blood pressure, HbA1c, HDL cholesterol, triglycerides, smoking status, and alcohol intake
**HDL-C**									
Avogaro et al. (40)	Italy	65	9,979 (51.7)	4	Patients with T2DM developed CHD events (MI, coronary artery bypass grafting, percutaneous transluminal coronary angioplasty, and electrocardiogram-proven angina)	881	Per 5 mg/dL higher	M: 0.98 (0.94–1.02) F: 0.96 (0.92–1.00)	Age, disease duration, serum triglycerides, microangiopathy, antihypertensive therapy, and insulin treatment, waist girth, glycemic control, total cholesterol, blood pressure, and geographic area and lipid-lowering
Sone et al. (62)	Japan	58.2	1,771 (46.9)	7.86 (median)	Patients with T2DM developed CHD (angina pectoris and MI)	109	Per 1 mmol/L	0.99 (0.56–1.74)	Gender, age, diabetes duration, body mass index, systolic blood pressure, HbA1c, LDL cholesterol, triglycerides, smoking status, and alcohol intake
**TGs**									
Protopsaltis et al. (49)	Greece	60.4	599 (46.0)	10.1 (median)	Patients with T2DM developed ischemic stroke	78	≥150 vs. < 150 mg/dL	1.03 (0.90–1.15)	Gender, age, smoking, body mass index, HbA1c, lipids, and diabetes duration
Sone et al. (43)	Japan	58.4	1,424 (45.9)	8	Patients with T2DM developed stroke;	59	≥150 vs. < 150 mg/dL	M: 1.10 (0.50–2.40) F: 0.70 (0.20–1.90);	NA
					Patients with T2DM developed CHD (MI, angina pectoris)	62		M: 2.90 (1.60–5.30) F: 1.70 (0.60–4.40)	
Avogaro et al. (40)	Italy	65	9,979 (51.7)	4	Patients with T2DM developed CHD events (MI, coronary artery bypass grafting, percutaneous transluminal coronary angioplasty, and electrocardiogram-proven angina)	881	≥150 vs. < 150 mg/dL	M: 1.19 (0.94–1.50) F: 1.33 (1.05–1.68)	Age, disease duration, microangiopathy, antihypertensive therapy, and insulin treatment, waist girth, glycemic control, total cholesterol, blood pressure, and geographic area, HDL cholesterol and lipid-lowering

CMM, cardiometabolic multimorbidity; FPG, fasting plasma glucose; LDL-C, low-density lipoprotein cholesterol; HDL-C, high-density lipoprotein cholesterol; TGs, triglycerides; T2DM, type 2 diabetes mellitus; IHD, ischemic heart disease; MI, myocardial infarction; CHD, coronary heart disease; HbA1c, hemoglobin A1c; HR, hazard ratio; CI, confidence interval; M, male; F, female; NA, not available.

A total of 8 studies were from the USA, followed by Japan (*n* = 7) and the UK (*n* = 6). The sample size varied considerably among included studies, ranging from 224 to 891,095 participants. The mean age of participants ranged from 46.9 to 72.5 years across included studies. Most studies were of high quality (*n* = 41), and one study was of moderate quality (Supplementary Figure S1A). The items “Ascertainment of exposure” and “Demonstration that outcome of interest was not present at start of study” were rated as low risk of bias in all included studies, and the item “Adequacy of follow up of cohorts” was rated as high risk in the most of studies (*n* = 28) (Supplementary Figure S1B).

### 3.2 Overview of biomarkers

**Figure 2:**
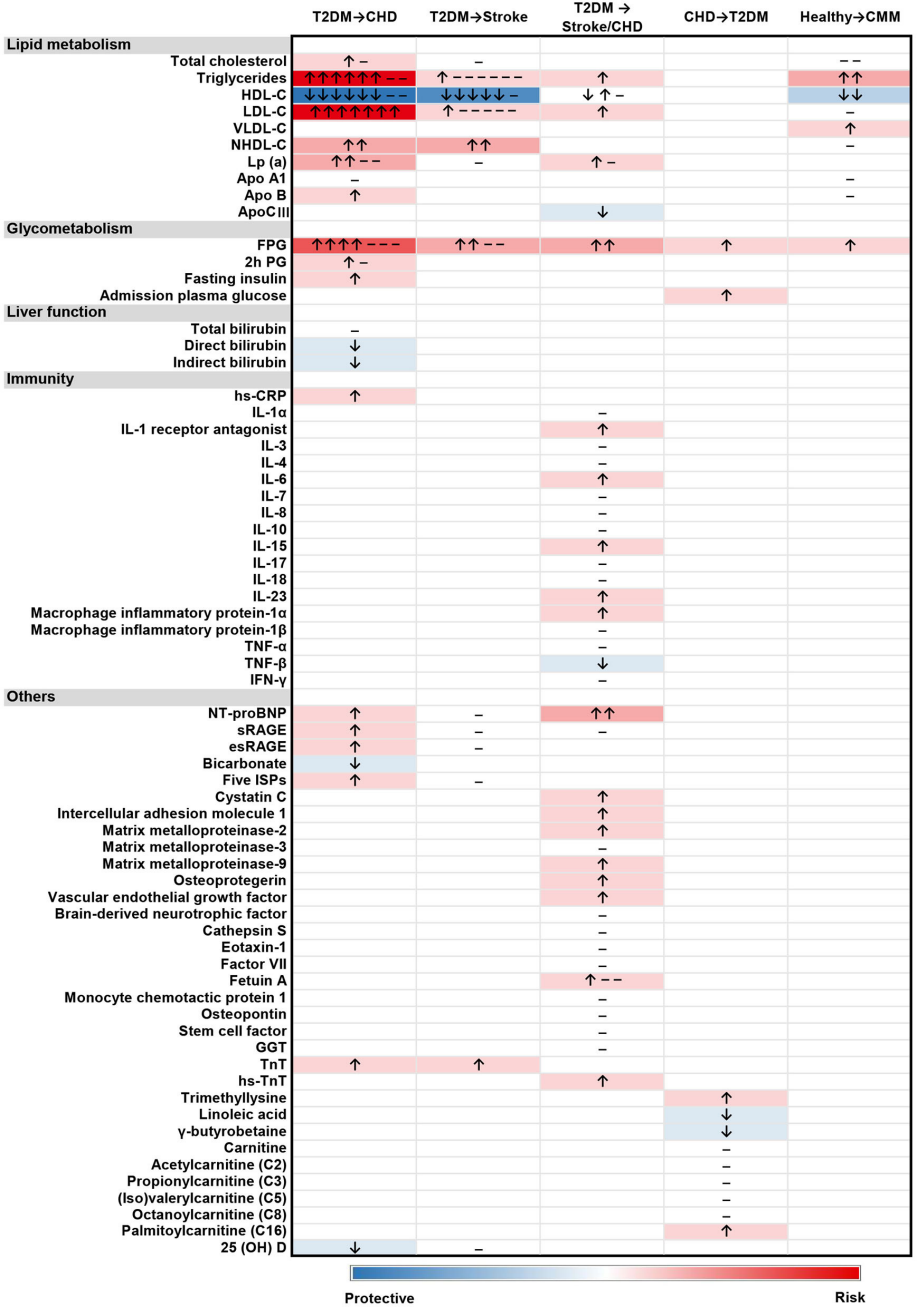
Heatmap of the associations between 68 biomarkers and 5 types of CMM progression. “↑”, positive association; “↓”, negative association; “–”, no significant association. The studies reporting different associations were counted and presented in the heatmap. The intensity of the color depended on the strength of overall association between biomarker level and the outcome.

Among the included studies, a total of 68 serum/plasma biomarkers were identified (Figure 2). They are categorized into five groups: lipid metabolism, glycometabolism, liver function, immunity, and others. The most frequently studied biomarkers were HDL-C (*n* = 19), TGs (*n* = 18), FPG (*n* = 15), LDL-C (*n* = 15), and lipoprotein-A [Lp(a)] (*n* = 7). Effects of some biomarkers reported in more than two studies were also presented according to races (Supplementary Figure S2). The directions of the association for most biomarkers with CMM were similar among different populations, except for HDL-C, which showed positive association with CMM in Chinese populations and negative association in Japanese. Four biomarkers (i.e., FPG, HDL-C, LDL-C, and TGs) were available for meta-analysis.

### 3.3 The role of biomarkers in different progression patterns of CMM

#### 3.3.1 Healthy participants progressed to CMM

Three studies (7, 66, 67) reported the association of lipid metabolism related biomarkers with the progression of CMM, including total cholesterol (TC), TGs, HDL-C, LDL-C, VLDL-C, NHDL-C, Apo A1, and ApoB. According to these studies, a higher level of TGs or VLDL-C was reported to positively associate with an increased risk of CMM, while HDL-C level was reported to inversely associate with CMM. FPG was the only glycometabolism biomarker associated with CMM, and a higher FPG level was positively associated with an increased risk of CMM.

#### 3.3.2 T2DM followed by CHD

**Figure 3:**
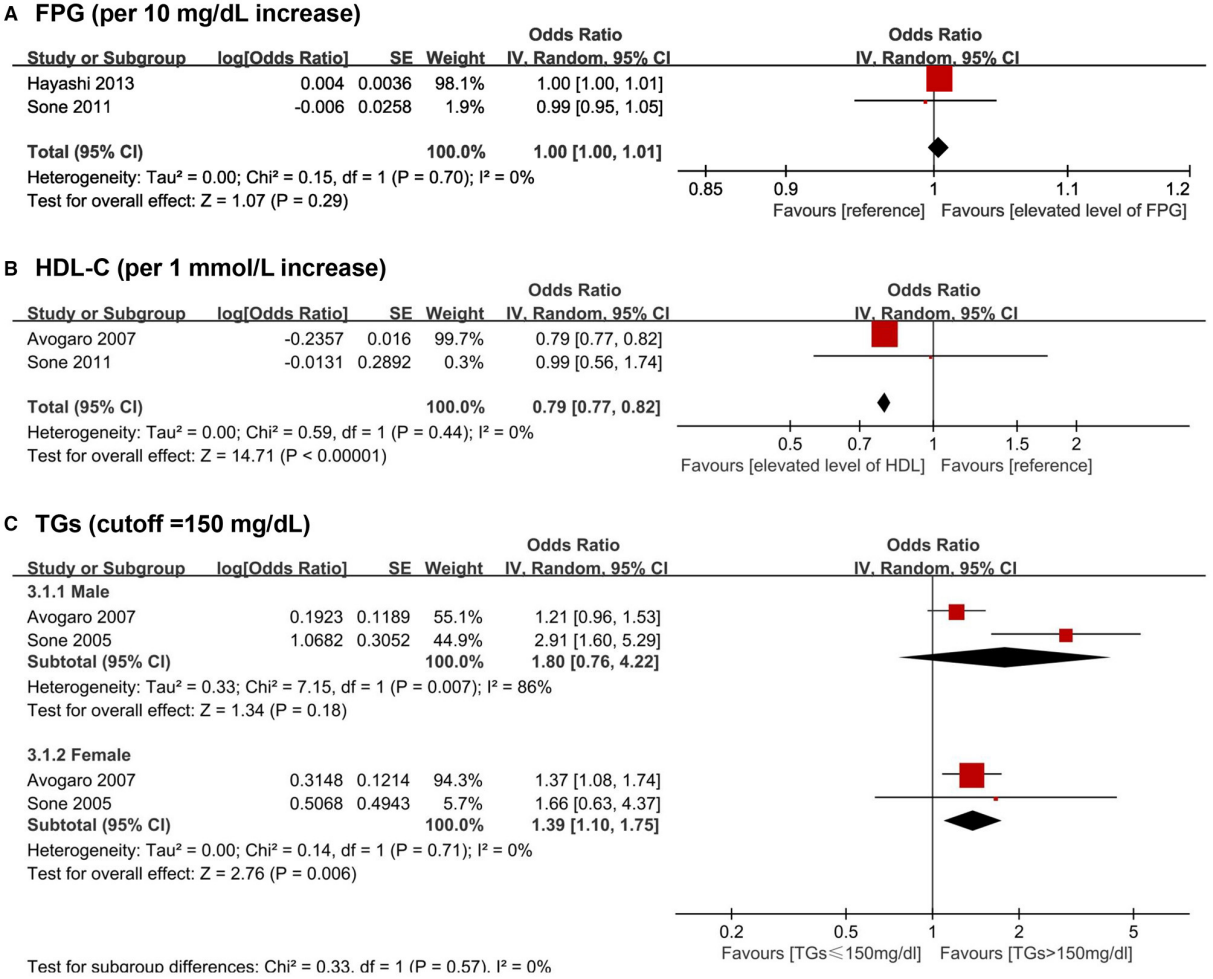
Forest plot of prospective studies examining FPG **(A)**, HDL-C **(B)**, TGs **(C)** levels and risk of CHD in subjects with T2DM. **(A)** Pooled analysis of increase risk of CHD in subjects with T2DM for per 10 mg/dL increase of FPG level. **(B)** Pooled analysis of increase risk of CHD in subjects with T2DM for per 1 mmol/L increase of HDL-C level. **(C)** 3.1.1 Pooled analysis of increase risk of CHD in male T2DM patients with TGs level higher than 150 mg/dL. **(C)** 3.1.2 Pooled analysis of increase risk of CHD in female T2DM patients with TGs level higher than 150 mg/dL. CHD, coronary heart disease; CI, confidence interval; FPG, fasting plasma glucose; HDL-C, high-density lipoprotein cholesterol; T2DM, type 2 diabetes mellitus; TGs, triglycerides.

A total of 15 biomarkers were reported to be positively associated with a higher risk of CHD among T2DM patients, including six lipid metabolism biomarkers [i.e., TC, TGs, LDL-C, NHDL-C, Lp(a), and Apo B], three glycometabolism biomarkers (i.e., FPG, 2h PG and fasting insulin), one immunity biomarkers (i.e., hs-CRP), and five other biomarkers [i.e., N-terminal pro-B type natriuretic peptide (NT-proBNP), sRAGE, esRAGE, five inflammation-sensitive plasma proteins (ISPs) and TnT]. Five biomarkers were reported to negatively associate with the risk of CHD following T2DM, including HDL-C, direct bilirubin, indirect bilirubin, bicarbonate, and 25(OH)D. Four articles (40, 43, 48, 62) were available for the meta-analyses, which showed no significant associations of FPG (pooled OR for per 10 mg/dL increase = 1.00, 95% CI = 1.00–1.01) with CHD among T2DM patients. A higher level of TGs was positively associated with CHD risk among female T2DM patients (pooled OR for higher than 150 mg/dL among male T2DM patients = 1.80, 95% CI = 0.76–4.22, pooled OR for higher than 150 mg/dL among female T2DM patients = 1.39, 95%CI: = 1.10–1.75). Besides, an inverse association of HDL-C (pooled OR for per 1 mmol/L increase = 0.79, 95% CI = 0.77–0.82) with CHD risk among T2DM patients was found (Figure 3).

#### 3.3.3 T2DM followed by stroke

**Figure 4:**
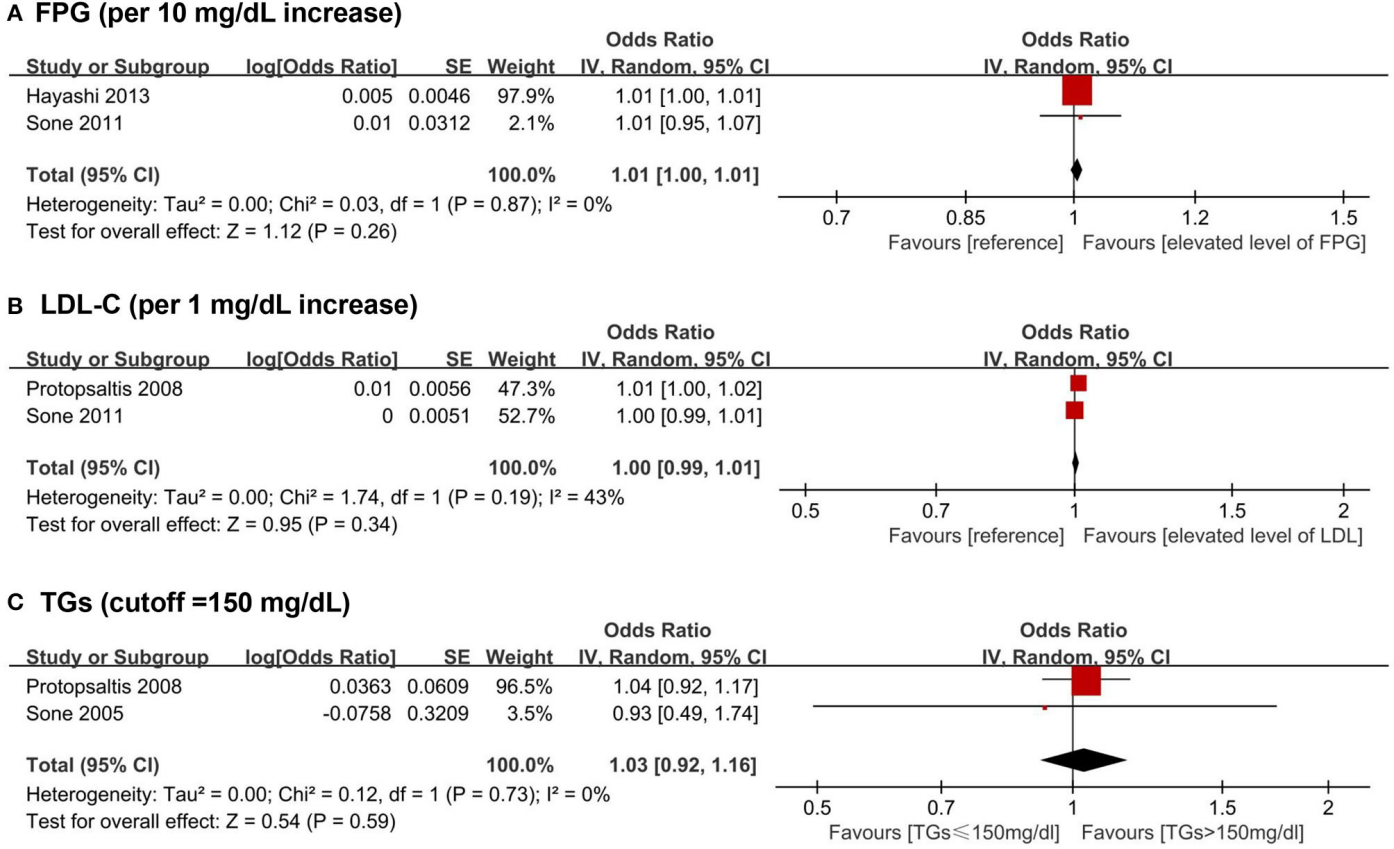
Forest plot of prospective studies examining FPG **(A)**, LDL-C **(B)**, TGs **(C)** levels and risk of stroke in subjects with T2DM. **(A)** Pooled analysis of increase risk of stroke in subjects with T2DM for per 10 mg/dL increase of FPG level. **(B)** Pooled analysis of increase risk of stroke in subjects with T2DM for per 1 mg/dL increase of LDL-C level. **(C)** Pooled analysis of increase risk of stroke in subjects with T2DM with TGs level higher than 150 mg/dL. CI, confidence interval; FPG, fasting plasma glucose; LDL-C, low-density lipoprotein cholesterol; T2DM, type 2 diabetes mellitus; TGs, triglycerides.

A total of five biomarkers were reported to be positively associated with a higher risk of stroke among T2DM patients, including three lipid metabolism biomarkers (i.e., LDL-C, TGs, and NHDL-C), one glycometabolism biomarker (i.e., FPG), and one other biomarker (i.e., TnT). Besides, HDL-C was reported to be inversely associated with the risk of stroke among the population with T2DM. Four articles (43, 48, 49, 62) were available for the meta-analyses: FPG (pooled OR for 10 mg/dL increase = 1.01, 95% CI = 1.00–1.01), LDL-C (pooled OR for 1 mg/dL increase = 1.00, 95% CI = 0.99–1.01), and TGs (pooled OR for higher than 150 mg/dL cutoff = 1.03, 95% CI = 0.92–1.16) showed no significant association with stroke among T2DM patients (Figure 4).

#### 3.3.4 T2DM followed by stroke or CHD

A total of 18 biomarkers were identified to be positively associated with a higher risk of stroke or CHD among T2DM patients, including three lipid metabolism biomarkers (i.e., Lp(a), LDL-C, and TGs), one glycometabolism biomarker (i.e., FPG), five immunity biomarkers (i.e., IL-1 receptor antagonist, IL-6, IL-15, IL-23, and macrophage inflammatory protein-1α) and nine other biomarkers [i.e., NT-proBNP, cystatin C, intercellular adhesion molecule 1 (ICAM-1), matrix metalloproteinase-2 (MMP-2), matrix metalloproteinase-9 (MMP-9), osteoprotegerin (OPG), vascular endothelial growth factor (VEGF), fetuin-A, and hs-TnT]. Besides, two biomarkers were inversely associated with the risk of stroke or CHD in T2DM patients, including apolipoprotein C III (Apo CIII) and tumor necrosis factor-β (TNF-β).

#### 3.3.5 CHD followed by T2DM

Two glycometabolism biomarkers (i.e., FPG and admission plasma glucose) and two additional biomarkers [trimethyllysine and palmitoylcarnitine (C16)] were reported to positively associated with a greater risk of T2DM in CHD patients. In addition, linoleic acid and γ-butyrobetaine were revealed to be inversely related to a greater risk of T2DM among CHD patients.

## 4 Discussion

### 4.1 Principle findings

We obtained 68 types of serum/plasma biomarkers from 42 included studies and summarized the evidence of associations between these biomarkers and five progression patterns of CMM. Lipid metabolism biomarkers were most reported to associate with the risk of CMM, including TC, TGs, HDL-C, LDL-C, and Lp(a). Only four biomarkers (FPG, HDL-C, LDL-C, TGs) were available for meta-analysis due to methodological heterogeneity among studies. A higher level of HDL-C was shown to significantly associate with lower risks of CHD among T2DM patients; on the other hand, a higher level of TGs was positively associated with CHD risk among female T2DM patients.

### 4.2 Comparison with previous studies

Lipid metabolism biomarkers were the most studied type of biomarkers. The meta-analysis showed that HDL-C was negative associated with CHD risk among patients with T2DM, and a higher TGs level was positively associated with CHD risk among female patients with T2DM.

Among all included studies, our heatmap suggested that elevated levels of TGs, LDL-C, NHDL-C, and Lp(a) were associated with a higher risk of CMM, while HDL-C presented a significant inverse association with the progression of CMM. In line with our findings, strong associations between lipid metabolism biomarkers (e.g., elevated levels of LDL-C, TGs and Lp(a), decreased level of HDL-C) and CMM have been found, especially in patients with T2DM (18, 19, 68–70). Also, it is worth noting that T2DM patients with high HDL-cholesterol levels had paradoxically higher risk of composite CVD outcomes in an included study (33). Previous studies (71, 72) also suggested that the association between HDL-C concentrations and CVD events might be a U-shaped curve, indicating that abnormally low or high HDL-C levels were both inversely associated with health status. However, due to the limited number and heterogeneity of included studies, we could not identify such an association in our analysis. There also existed sex differences in the effect of lipid metabolism biomarkers on the risk of coronary heart disease in patients with T2DM. One included study (40) reported the risk effect of TGs, and the protective effect of HDL-C on CHD only existed in female participants. Another study (43) showed a significant association between high levels of LDL-C and CHD outcome only in males. It can be explained that women with T2DM were more susceptible to the atherogenic effects of non-LDL-C factors (i.e., high level of TGs and low level of HDL-C) (73, 74). Future studies are warranted to validate such associations.

Several glycometabolism biomarkers were also reported to associate with CMM, especially for FPG. Hyperglycemia and uncontrolled glycemia have been extensively discussed as risk factors for the development of stroke and CHD in previous studies (20, 75, 76) in both the general population and T2DM patients. Hyperglycemia can detrimentally affect normal endothelial function, contributing to plaque formation and rupture, and finally, thrombosis (76, 77), thus correlating with a higher risk of atherosclerotic cardiovascular diseases (ASCVD). In addition, a J-shaped relationship between FPG and adverse cardiovascular events has been reported in people with T2DM (78, 79), especially in older patients with high comorbidity load, consistent with our results (54, 63). As a traditional diagnostic biomarker for T2DM, the level of FPG may provide more information for health status that could also serve as an indicator of the deterioration of T2DM and a potential biomarker for CMM.

Several inflammatory factors were also identified to associate with CMM in our study. In addition to what we found, many previous studies reported that an elevated concentration of inflammatory factors (e.g., IL-6, hs-CRP, TNF-α) were positively correlated with ASCVD outcomes (80–82) due to their effects on plaque development and rupture, endothelial dysfunction, and coronary thrombosis (82). However, the role of some other immunity factors in the progression of CVD is controversial, and there remains a lack of evidence to assess the association between immunity biomarkers and the development of CMM.

The majority of the immunity biomarkers in our research were collected from one study (57), which intended to choose predictive biomarkers for CVD in T2DM patients. More research is needed to determine the relevance of immunity biomarkers as potential indications of CMM progression.

Liver function related biomarkers were also crucial in the progression of CMM, but current findings of their impacts on CMM were scarce and inconsistent. For example, bilirubin, an essential marker of liver function, has been indicated as an antioxidant with anti-inflammatory and antiapoptotic effects (83–85). One included study (60) revealed that higher levels of serum direct and indirect bilirubin were related to decreased CHD risk in T2DM patients. But the evidence from prospective studies was limited. Another study (86) suggested that serum bilirubin could add predictive value to future cardiovascular deaths in patients with T2DM. Besides, NT-proBNP was widely used in detecting heart failure and was also a prognostic marker in patients with acute decompensated heart failure (87, 88). Another review (89) regarded NT-proBNP as a predictor of CVD events in T2DM patients, which was consistent with our findings.

Many included studies recruited patients with one initial CMD at baseline and most had T2DM. It has been proved that hypertension and dyslipidemia were quite common in patients with T2DM, even in prediabetic individuals (90–92). Many studies reported using current diabetes treatments, antihypertensive therapy, and lipid-lowering treatments at baseline, which could interfere with the biomarker measurements and the association between biomarkers and CMD (93, 94). Several studies addressed the influence of medication and incorporated it into the multivariate model. Some studies compared the risk effect before and after further adjustment for treatment and observed similar results. However, the impact of treatment and drugs was not accounted for in some studies due to the complexity of potential interactions. The role of medication in the association between biomarkers and CMM requires further investigation to prevent the development of CMM in people who already have one basis disease.

Lifestyle and diet habits are also important factors associated with certain biomarkers levels and CMM progression according to previous studies (6, 12, 95–97). Many included studies adjusted alcohol intake and physical activity as covariates in the multivariate analysis. However, few of them estimated the effect sizes of these factors with biomarkers or further discuss other lifestyle or diet factors. The interaction between lifestyle, diet habits, biomarkers, and CMM awaits more investigations and explorations in the future.

### 4.3 Implications

For future research, there remains a lack of observational studies, especially longitudinal cohort studies, to provide more evidence on the associations between biomarkers and the progression of CMM. Evidence on individuals from an apparently healthy state to CMM and various CMM patterns is needed since the majority of included studies in our review focused on populations with T2DM at baseline. Also, the role and effect of medications, treatment, lifestyle, and diet factors in the association between biomarkers and cardiometabolic diseases await more evidence and further exploration. Furthermore, it is also worth exploring the variation of biomarkers over time in the progression of CMM. Finally, a few included studies reported the joint assessments of multiple biomarkers, but the prediction performance of multiple biomarkers in comparison with a single biomarker still needs more evidence.

Our results highlight the value of serum/plasma biomarkers in the primary prevention of CMM among healthy people and secondary prevention in people who already have CMD. Lowering levels of risk biomarkers could be considered potential preventive targets of lifestyle and therapeutic interventions. Our finding may also provide important clinical implications for the early screening and prediction of CMM through targeted biomarkers. Measurements of serum biomarkers in the general population may help to identify individuals at high risk and maximize healthcare resources.

### 4.4 Limitations

Some limitations of our current review should be addressed. First, because of the limited number of available studies, several biomarkers were only reported once or twice, suggesting that there may be publication bias. Additionally, most included studies were conducted in specific regions or countries, recruiting participants of specific race. Previous studies have shown the various clinical impacts of some metabolic characteristics [e.g., metabolic syndrome (43), serum triglyceride levels (62)] on diabetes across different races. The results could not be fully generalized to the overall population. Third, although many studies included potential confounders in the multivariate models, the adjustments differed in the originally included studies, and some studies' confounders were unavailable. Finally, as mentioned above, integrative analysis of some biomarkers failed due to methodological heterogeneity across studies, which may lead to unconvincing results of the meta-analysis.

## 5 Conclusion

Our systematic review and meta-analysis summarized the evidence on the role of a broad number of biomarkers in the development and progression of CMM. However, studies focusing on the association of biomarkers and CMM were scarce, requesting more evidence on this topic to provide implications for early prevention, detection, and intervention of CMM.

## Data availability statement

The original contributions presented in the study are included in the article/Supplementary material, further inquiries can be directed to the corresponding author.

## Author contributions

XX: Conceptualization, Methodology, Project administration, Supervision, Validation, Writing—review & editing. YJ: Data curation, Formal analysis, Investigation, Methodology, Validation, Visualization, Writing—original draft. ZX: Data curation, Formal analysis, Investigation, Methodology, Validation, Writing—original draft. YutZ: Data curation, Investigation, Methodology, Validation, Writing—original draft. YueZ: Writing—review & editing. DW: Methodology, Visualization, Writing—review & editing. YC: Writing—review & editing. YZho: Writing—review & editing. MF: Writing—review & editing.
