# Supplementary material for: Serum/plasma biomarkers and the progression of cardiometabolic multimorbidity: a systematic review and meta-analysis
Source: Front Public Health. 2023 Nov 23;11:1280185. doi: 10.3389/fpubh.2023.1280185 (PMC10701686; doi:10.3389/fpubh.2023.1280185)
Supplement: Supplementary file 1 [file Data_Sheet_1.docx]

Supplementary Material

# Supplementary Figures and Tables

## Supplementary Figures

Supplementary Figure 1. Risk of bias summary (A) & graph (B).


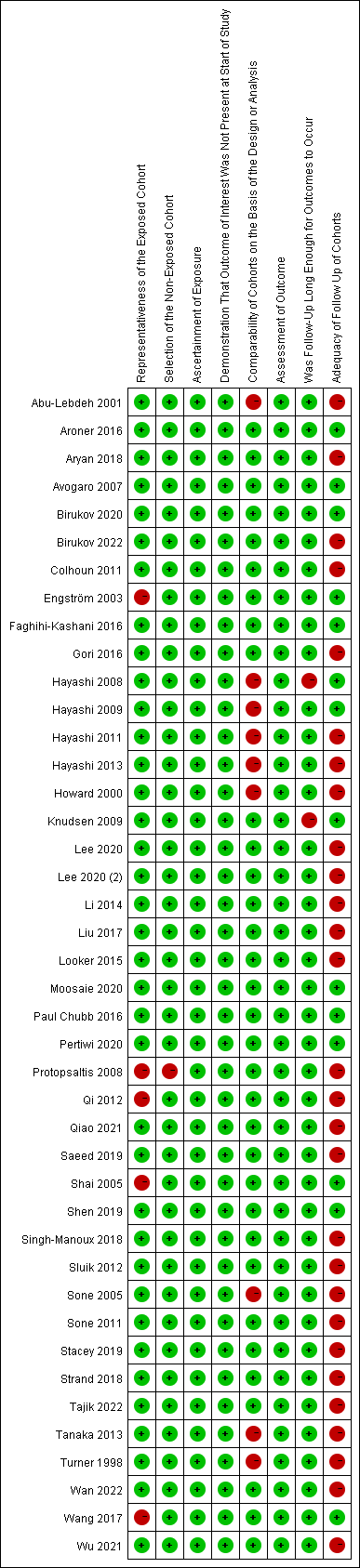


1. Red means the item was scored “0”, and Green means scored “1”.


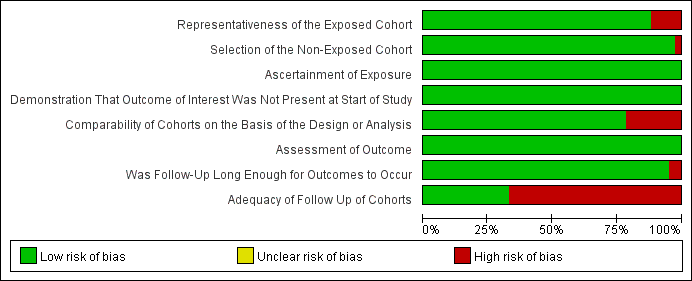


1. Judgements about each risk of bias item presented as percentages across all included studies.


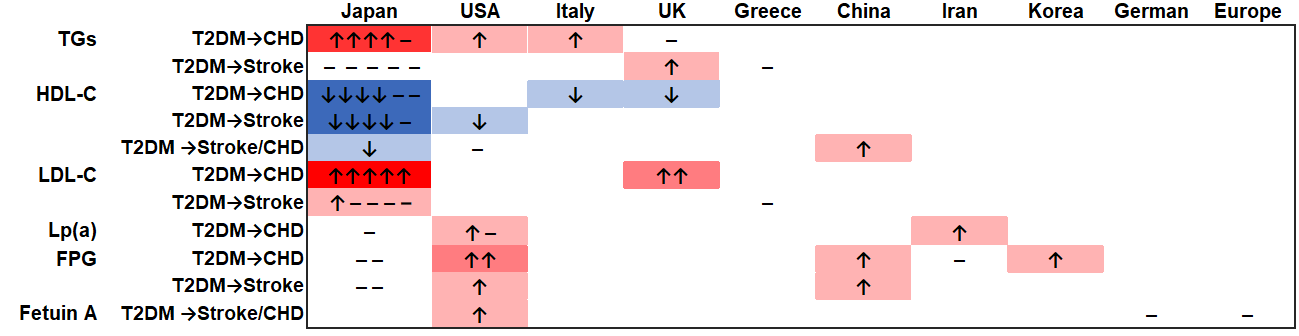


**Supplementary Figure 2.** Heatmap of the associations between biomarkers and CMM progression according to races.

Note: “↑”, positive association; “↓”, negative association; “−”, no significant association. The studies reporting different associations were counted and presented in the heatmap. The intensity of the color depended on the strength of overall association between biomarker level and the outcome.

## Supplementary Tables

**Table S1** Literature search strategy

PubMed

| No. | Query |
| --- | --- |
| #1 | "multimorbidity"[MeSH Major Topic] OR "multimorbidit*"[Title] OR "multi-morbidit*"[Title] OR "comorbidit*"[Title] OR "co-morbidit*"[Title] OR "multiple chronic diseases*"[Title] |
| #2 | "cardio metabol*"[Title/Abstract] OR "cardio-metabol*"[Title/Abstract] OR "cardiometabol*"[Title/Abstract] OR "cardio*"[Title/Abstract] |
| #3 | #1 AND #2 |
| #4 | "stroke"[MeSH Major Topic] OR "stroke*"[Title/Abstract] OR "cerebrovasc*"[Title/Abstract] OR "brain vasc*"[Title/Abstract] OR "vascular accident*"[Title/Abstract] |
| #5 | "diabetes mellitus"[MeSH Major Topic] OR "diabet*"[Title/Abstract] |
| #6 | "cardiovascular diseases"[MeSH Major Topic] OR "cardio*"[Title/Abstract] OR "myocardial*"[Title/Abstract] OR "coronary disease*"[Title/Abstract] OR "myocardial infarct*"[Title/Abstract] OR "myocardial ischemia"[Title/Abstract] OR "heart attack*"[Title/Abstract] OR "angina pectoris"[Title/Abstract] OR "angor pectoris"[Title/Abstract] OR "stenocardia*"[Title/Abstract] OR "transient ischemic attack*"[Title/Abstract] |
| #7 | ("atheroscleros*"[Title/Abstract] OR "atherogenes*"[Title/Abstract]) AND ("coronary artery"[Title/Abstract] OR "cardio*"[Title/Abstract]) |
| #8 | #6 OR #7 |
| #9 | #4 AND #5 |
| #10 | #4 AND #8 |
| #11 | #5 AND #8 |
| #12 | #4 AND #5 AND #8 |
| #13 | #3 OR #9 OR #10 OR #11 OR #12 |
| #14 | "biomarkers"[MeSH Major Topic] OR "marker*"[Title/Abstract] OR "biomarker*"[Title/Abstract] OR "signature molecule"[Title/Abstract] OR "molecular marker*"[Title/Abstract] OR "biological"[Title/Abstract] OR "serum"[Title/Abstract] OR "plasma"[Title/Abstract] OR "C-reactive protein"[Title/Abstract] OR "CRP"[Title/Abstract] OR "triglycerides"[Title/Abstract] OR "creatinine"[Title/Abstract] OR "blood urea nitrogen"[Title/Abstract] OR "BUN"[Title/Abstract] OR "glucose"[Title/Abstract] OR "total cholesterol"[Title/Abstract] OR "high-density lipoprotein"[Title/Abstract] OR "HDL"[Title/Abstract] OR "low-density lipoprotein"[Title/Abstract] OR "LDL"[Title/Abstract] OR "uric acid"[Title/Abstract] OR "cystatin c"[Title/Abstract] |
| #15 | #13 AND #14 |
| #16 | "case-control"[Title/Abstract] OR "cohort*"[Title/Abstract] OR "prospective"[Title/Abstract] OR "cross-sectional"[Title/Abstract] OR ("population-based"[Title] AND "study"[Title]) |
| #17 | "humans"[MeSH Terms] AND "English"[Language] |
| #18 | #15 AND #16 AND #17 |

Ebsco

| No. | Query |
| --- | --- |
| #1 | TI(multimorbidit* OR multi-morbidit* OR comorbidit* OR co-morbidit* OR "multiple chronic diseases*") |
| #2 | TI(“cardio metabol*” OR cardio-metabol* OR cardiometabol* OR cardio*) |
| #3 | AB(“cardio metabol*” OR cardio-metabol* OR cardiometabol* OR cardio*) |
| #4 | S2 OR S3 |
| #5 | S1 AND S4 |
| #6 | TI(stroke* OR cerebrovasc* OR brain vasc* OR vascular accident*) |
| #7 | AB(stroke* OR cerebrovasc* OR brain vasc* OR vascular accident*) |
| #8 | S6 OR S7 |
| #9 | TI(diabet*) |
| #10 | AB(diabet*) |
| #11 | S9 OR S10 |
| #12 | TI( cardio* OR myocardial* OR (coronary N3 disease*) OR (("atheroscleros*" OR "atherogenes*") AND("coronary artery" OR "cardio*")) OR “myocardial infarct*” OR “myocardial ischemia” OR “heart attack*” OR “angina pectoris” OR “angor pectoris” OR stenocardia* OR (transient N3 ischem* N3 attack*)) |
| #13 | AB( cardio* OR myocardial* OR (coronary N3 disease*) OR (("atheroscleros*" OR "atherogenes*") AND("coronary artery" OR "cardio*")) OR “myocardial infarct*” OR “myocardial ischemia” OR “heart attack*” OR “angina pectoris” OR “angor pectoris” OR stenocardia* OR (transient N3 ischem* N3 attack*)) |
| #14 | S12 OR S13 |
| #15 | S8 AND S11 |
| #16 | S8 AND S14 |
| #17 | S11 AND S14 |
| #18 | S17 AND S16 AND S15 |
| #19 | TI( marker* OR biomarker* OR “signature molecule” OR “molecular marker*” OR biological OR serum OR plasma OR “C-reactive protein” OR CRP OR triglycerides OR creatinine OR “blood urea nitrogen” OR BUN OR glucose OR “total cholesterol” OR “high-density lipoprotein” OR HDL OR “low-density lipoprotein” OR LDL OR “uric acid” OR “cystatin c”) |
| #20 | AB( marker* OR biomarker* OR “signature molecule” OR “molecular marker*” OR biological OR serum OR plasma OR “C-reactive protein” OR CRP OR triglycerides OR creatinine OR “blood urea nitrogen” OR BUN OR glucose OR “total cholesterol” OR “high-density lipoprotein” OR HDL OR “low-density lipoprotein” OR LDL OR “uric acid” OR “cystatin c”) |
| #21 | S19 OR S20 |
| #22 | S18 OR S17 OR S16 OR S15 OR S5 |
| #23 | S21 AND S22 |
| #24 | TI(case-control OR cohort* OR prospective OR cross-sectional) |
| #25 | AB(case-control OR cohort* OR prospective OR cross-sectional) |
| #26 | TI(population-based AND study) |
| #27 | S24 OR S25 OR S26 |
| #28 | S23 AND S27 |
| #29 | S28 Limiters – Full Text; English Language; Research Article; Human |

Web of Science

| No. | Query |
| --- | --- |
| #1 | TI=(multimorbidit* OR multi-morbidit* OR comorbidit* OR co-morbidit* OR "multiple chronic diseases*") |
| #2 | TI=(“cardio metabol*” OR cardio-metabol* OR cardiometabol* OR cardio*) |
| #3 | AB=(“cardio metabol*” OR cardio-metabol* OR cardiometabol* OR cardio*) |
| #4 | #2 OR #3 |
| #5 | #1 AND #4 |
| #6 | TI=(stroke* OR cerebrovasc* OR brain vasc* OR vascular accident*) |
| #7 | AB=(stroke* OR cerebrovasc* OR brain vasc* OR vascular accident*) |
| #8 | #6 OR #7 |
| #9 | TI=diabet* |
| #10 | AB=diabet* |
| #11 | #9 OR #10 |
| #12 | TI=( cardio* OR myocardial* OR (coronary NEAR/3 disease*) OR (("atheroscleros*" OR "atherogenes*") AND("coronary artery" OR "cardio*")) OR “myocardial infarct*” OR “myocardial ischemia” OR “heart attack*” OR “angina pectoris” OR “angor pectoris” OR stenocardia* OR (transient NEAE/3 ischem* NEAR/3 attack*)) |
| #13 | AB=( cardio* OR myocardial* OR (coronary NEAR/3 disease*) OR (("atheroscleros*" OR "atherogenes*") AND("coronary artery" OR "cardio*")) OR “myocardial infarct*” OR “myocardial ischemia” OR “heart attack*” OR “angina pectoris” OR “angor pectoris” OR stenocardia* OR (transient NEAE/3 ischem* NEAR/3 attack*)) |
| #14 | #12 OR #13 |
| #15 | #8 AND #11 |
| #16 | #8 AND #14 |
| #17 | #11 AND #14 |
| #18 | #17 AND #16 AND #15 |
| #19 | TI=( marker* OR biomarker* OR “signature molecule” OR “molecular marker*” OR biological OR serum OR plasma OR “C-reactive protein” OR CRP OR triglycerides OR creatinine OR “blood urea nitrogen” OR BUN OR glucose OR “total cholesterol” OR “high-density lipoprotein” OR HDL OR “low-density lipoprotein” OR LDL OR “uric acid” OR “cystatin c”) |
| #20 | AB=( marker* OR biomarker* OR “signature molecule” OR “molecular marker*” OR biological OR serum OR plasma OR “C-reactive protein” OR CRP OR triglycerides OR creatinine OR “blood urea nitrogen” OR BUN OR glucose OR “total cholesterol” OR “high-density lipoprotein” OR HDL OR “low-density lipoprotein” OR LDL OR “uric acid” OR “cystatin c”) |
| #21 | #19 OR #20 |
| #22 | #18 OR #17 OR #16 OR #15 OR #5 |
| #23 | #21 AND #22 |
| #24 | TI=(case-control OR cohort* OR prospective OR cross-sectional) |
| #25 | AB=(case-control OR cohort* OR prospective OR cross-sectional) |
| #26 | TI=(population-based AND study) |
| #27 | #24 OR #25 OR #26 |
| #28 | #23 AND #27 |
| #29 | #28 language:(English) AND literature type: (Article) |

Embase

| No. | Query |
| --- | --- |
| #1 | 'multiple chronic conditions'/exp |
| #2 | multimorbidit*:ti OR 'multi-morbidit*':ti OR comorbidit*:ti OR 'co-morbidit*':ti OR 'multiple chronic disease*':ti |
| #3 | #1 OR #2 |
| #4 | 'cardio metabol*':ti,ab OR cardio-metabol*:ti,ab OR cardiometabol*:ti,ab OR cardio*:ti,ab |
| #5 | #3 AND #4 |
| #6 | 'cerebrovascular accident'/exp |
| #7 | 'stroke*':ti,ab OR 'cerebrovasc*':ti,ab OR 'brain vasc*':ti,ab OR 'vascular accident*':ti,ab |
| #8 | #6 OR #7 |
| #9 | 'diabetes mellitus'/exp |
| #10 | 'diabet*':ti,ab |
| #11 | #9 OR #10 |
| #12 | #8 AND #11 |
| #13 | 'cardiovascular disease'/exp |
| #14 | cardio*:ab,ti OR 'myocardial*':ti,ab OR ((coronary NEAR/3 disease*):ti,ab) OR (('atheroscleros*':ti,ab OR 'atherogenes*':ti,ab) AND ('coronary artery':ti,ab OR 'cardio*':ti,ab)) OR 'myocardical infarct*':ti,ab OR 'myocardial ischemia':ti,ab OR 'heart attack*':ti,ab OR 'angina pectoris':ti,ab OR 'angor pectoris':ti,ab OR stenocardia*:ti,ab OR ((transient NEAR/3 ischem* NEAR/3 attack*):ti,ab) |
| #15 | #13 OR #14 |
| #16 | #11 AND #15 |
| #17 | #8 AND #15 |
| #18 | #8 AND #11 AND #15 |
| #19 | #5 OR #12 OR #16 OR #17 OR #18 |
| #20 | 'biological marker'/exp |
| #21 | marker*:ab,ti OR biomarker*:ab,ti OR 'signature molecule':ab,ti OR 'molecular marker*':ab,ti OR biological:ab,ti OR serum:ab,ti OR plasma:ab,ti OR 'c-reactive protein':ab,ti OR crp:ab,ti OR triglycerides:ab,ti OR creatinine:ab,ti OR 'blood urea nitrogen':ab,ti OR bun:ab,ti OR glucose:ab,ti OR 'total cholesterol':ab,ti OR 'high-density lipoprotein':ab,ti OR hdl:ab,ti OR 'low-density lipoprotein':ab,ti OR ldl:ab,ti OR 'uric acid':ab,ti OR 'cystatin c':ab,ti |
| #22 | #20 OR #21 |
| #23 | #19 AND #22 |
| #24 | 'case-control':ab,ti OR cohort*:ab,ti OR prospective:ab,ti OR 'cross-sectional':ab,ti |
| #25 | 'population-based':ti AND study:ti |
| #26 | #24 OR #25 |
| #27 | #23 AND #26 |
| #28 | #23 AND #26 AND [article]/lim AND [english]/lim AND [humans]/lim AND [embase]/lim |

**Table S2** Characteristics of the included studies

| **Study** | **Study design** | **Country** | **Population** | **Age at baseline (mean ± SD)** | **Sex distribution (female%)** | **Sample size** | **Follow-up years** | **Biomarkers** | **Definition of CMM** | **No. of events** | **Comparison** | **Outcome/HR or RR or OR (95%CI)** | **Covariates** |
| --- | --- | --- | --- | --- | --- | --- | --- | --- | --- | --- | --- | --- | --- |
| ***DM→CHD*** | |  |  |  |  |  |  |  |  |  |  |  |  |
| Abu-Lebdeh 2001 | Cohort study | USA | Patients with T2DM | 57 ± 12 | 53 | 449 | 13 (mean) | TGs | Coronary artery disease (angina pectoris, myocardial infarction, and CAD deaths) | 216 | Per 1 natural log unit | HR 1.49 (1.15-1.92) | NA |
|  |  |  |  |  |  |  |  | FPG |  |  |  | HR 1.63 (1.17-2.25) |  |
| Aryan 2018 | Cohort study | Iran | Patients with T2DM | 55.1 ± 0.3 | 52.3 | 1,301 | 7.5 (mean) | hs-CRP | CHD events (non-fatal myocardial infarction, acute ischemic events requiring hospitalization and revascularization procedures, and CHD-related death) | 185 | Per 10% baseline hs-CRP | HR 1.028 (1.024-1.032) | Age, gender, HDL-c, LDL-c, BMI, waist circumference, smoking, SBP, lipid lowering and anti-hypertensive medications |
| Avogaro 2007 | Cohort study | Italy | Patients with T2DM | 65 | 51.66 | 9,979 | 4 | HDL-C | CHD events (myocardial infarction, coronary artery bypass grafting, percutaneous transluminal coronary angioplasty, and electrocardiogram-proven angina) | 881 | Per 5 mg/dL higher | HR 0.98 (0.94-1.02) M 0.96 (0.92-1.00) F | Age, disease duration, serum triglycerides, microangiopathy, antihypertensive therapy, and insulin treatment, waist girth, glycemic control, total cholesterol, blood pressure, and geographic area and lipid-lowering |
|  |  |  |  |  |  |  |  | TGs |  |  | ≥150 mg/dL vs. <150 mg/dL | HR 1.19 (0.94-1.50) M 1.33 (1.05-1.68) F | Age, disease duration, microangiopathy, antihypertensive therapy, and insulin treatment, waist girth, glycemic control, total cholesterol, blood pressure, and geographic area, HDL cholesterol and lipid-lowering |
|  |  |  |  |  |  |  |  | TC |  |  | Per 40 mg/dL higher | HR 1.03 (0.96-1.12) M 0.97 (0.87-1.08) F | Age, disease duration, serum triglycerides, microangiopathy, antihypertensive therapy, and insulin treatment, waist girth, glycemic control, blood pressure, and geographic area, HDL cholesterol and lipid-lowering |
| Colhoun 2011 | Nested case-control study | U.K. and Ireland | Patients with T2DM | 61.7 ± 1.3 | 28.6 | 718 | 3.9 (median) | sRAGE | CHD (myocardial infarction including silent infarction, unstable angina, acute coronary heart disease (CHD) death, resuscitated cardiac arrest, coronary revascularization procedures) | 128 | A doubling of the sRAGE level | HR 1.74 (1.25-2.41) | Age, sex, and treatment group, lipids, BMI, ethnicity, smoking, SBP, diabetes duration, and HbA2c, baseline eGFR and albuminuria |
|  |  |  |  |  |  |  |  | esRAGE |  |  | A doubling of the esRAGE level | HR 1.45 (1.11-1.89) |  |
| Engström 2003 | Cohort study | Sweden | Patients with T2DM | 46.9 | 0 | 321 | 18.7 ± 3.7 | Five ISPs (fibrinogen, orosomucoid, α1-antitrypsin, ceruloplasmin, and haptoglobin) | Cardiac event (fatal or nonfatal myocardial infarction or death due to chronic ischemic heart disease) | 30 | Two to five ISPs in top quartile vs. Zero to one ISP in top quartile | RR 2.2 (1.2–3.8) | Age, cholesterol, and ISP levels |
| Faghihi-Kashani 2016 | Cohort study | Iran | Patients with T2DM | 54.72 ± 11.16 | 55.78 | 2,607 | 7.2 (mean) | FPG | CHD event( myocardial infarction, angina pectoris, coronary insufficiency or death attributable to CHD) | 299 | Per one-quartile | HR 0.92 (0.8-1.06) | Gender, LDL-C, BMI, SBP, DBP, family history of CHD, smoking, medication use and HbA1c(mutually adjusted) |
|  |  |  |  |  |  |  |  | 2-hPG |  |  | Per one-quartile | HR 1.15 (1.00-1.32） |  |
|  |  |  |  |  |  |  |  | Fasting insulin |  |  | Per one-quartile | HR 1.18 (1.06-1.32) |  |
| Gori 2016 | Cohort study | America | Patients with T2DM | 63 | 52 | 1,510 | 13.1 (median) | NT-pro-BNP | CHD (definite fatal CHD, definite or probable MI, or coronary revascularization) | NA | Per 1 SD higher | HR 1.54 (1.16-2.03) | Age, sex, race, and field center, smoking status, log-transformed pack-years of cigarettes (packs of cigarettes smoked per day times number of years smoked), BMI, waist-to-hip ratio, systolic blood pressure, hypertension medication use, lipid-lowering medication use, aspirin use, education level, total-to-HDL cholesterol ratio, log-transformed triglycerides, and duration of diabetes, conventional measures of diabetes complications (retinopathy, nephropathy, PAD) and ECG abnormalities, cardiac biomarkers (abnormal TnT and abnormal NTproBNP) |
|  |  |  |  |  |  |  |  | TnT |  |  | Per 1 SD higher | HR 1.79 (1.34-2.39) |  |
| Hayashi 2009 | Prospective cohort study | Japan | Patients with T2DM | 67.4 ± 9.5 | NA | 4,014 | 2 | LDL-C | IHD (myocardial infarction, unstable angina pectoris, angioplasty, or bypass grafting) | NA | NA | OR 1.225 (1.02-2.04) <65Yr 1.001 (0.72-1.25) 65-74Yr 0.776 (0.43-1.40) >75Yr | Gender |
|  |  |  |  |  |  |  |  | HDL-C |  |  | NA | OR 0.659 (0.39-0.98) <65Yr 0.939 (0.68-1.25) 65-74Yr 0.946 (0.58-1.29) >75Yr |  |
|  |  |  |  |  |  |  |  | TGs |  |  | NA | OR 1.356 (1.00-2.02) <65Yr 0.731 (0.52-1.94) 65-74Yr 0.881 (0.46-1.70) >75Yr |  |
| Hayashi 2011 | Prospective cohort study | Japan | Patients with T2DM | 70.4 (median) | 49.85 | 4,014 | 2 | LDL-C | IHD (comprising fatal or nonfatal myocardial infarction; development of unstable angina; or the need for coronary revascularization procedures, either coronary artery bypass grafting or percutaneous coronary intervention because of angina or an acute coronary syndrome) | 82 | NA | OR 1.12 (1.00-1.28) <=70Yr, HgbA1C=<7.0  1.04 (0.87-1.21) <=70Yr, 7.0<HgbA1C  0.99 (0.78-1.11) >70Yr, HgbA1C=<7.0 1.05 (0.87-1.24) >70Yr, 7.0<HgbA1C | NA |
|  |  |  |  |  |  |  |  | HDL-C |  |  | NA | OR 0.46 (0.26-0.85) <=70Yr, HgbA1C=<7.0  0.78 (0.53-1.08) <=70Yr, 7.0<HgbA1C 0.99 (0.73-1.26) >70Yr, HgbA1C=<7.0  0.79(0.50-1.18) >70Yr, 7.0<HgbA1C |  |
|  |  |  |  |  |  |  |  | TGs |  |  | NA | OR 1.01 (0.99-1.03) <=70Yr, HgbA1C=<7.0  1.01 (0.94-1.04) <=70Yr, 7.0<HgbA1C 1.00 (1.00-1.09) >70Yr, HgbA1C=<7.0  1.01 (0.99-1.04) >70Yr, 7.0<HgbA1C |  |
| Hayashi 2013 | Prospective cohort study | Japan | Patients with T2DM | 67.4 ± 9.5 | 48.2 | 4,014 | 5.5 | LDL-C | IHD (Definite fatal and nonfatal myocardial infarction OR Angina pectoris) | 153 | Highest quartile vs. lowest quartile | HR 1.318 (1.103-1.585) total 1.571 (1.128-2.524) <65Yr 1.050 (0.932-1.176) 65-74Yr 1.156 (0.998-1.309) >75Yr | NA |
|  |  |  |  |  |  |  |  | HDL-C |  |  | Highest quartile vs. lowest quartile | HR 0.751 (0.611-0.917) total 0.828 (0.646-1.017) <65Yr 0.987 (0.966-1.008) 65-74Yr 0.629 (0.401-0.856) >75Yr |  |
|  |  |  |  |  |  |  |  | TGs |  |  | Highest quartile vs. lowest quartile | HR 1.005 (0.889-1.166) total 1.002 (0.996-1.006) <65Yr 1.108 (0.997-1.220) 65-74Yr 1.001 (0.961-1.046) >75Yr |  |
|  |  |  |  |  |  |  |  | Non-HDL-C |  |  | Highest quartile vs. lowest quartile | HR 1.023 (0.981-1.072) total 1.025 (1.001-1.121) <65Yr 1.073 (0.982-1.161) 65-74Yr 0.941 (0.791-1.102) >75Yr |  |
|  |  |  |  |  |  |  |  | FPG |  |  | Per 10 mg/dL higher | HR 1.004 (0.997-1.008) total 1.005 (0.996-1.013) <65Yr 1.004 (0.997-1.009) 65-74Yr 0.999 (0.987-1.007) >75Yr |  |
| Lee 2020 | Cohort study | Korea | Patients with T2DM | 40-64 | 36 | 891,095 | 7.1 ± 1.2 | TC | Incident myocardial infarction | 17,460 | 160-200 mg/dL vs.<160 mg/dL | HR 1.09 (1.04-1.14) | Age, sex, smoking, regular exercise, body mass index, chronic kidney disease, duration of diabetes, numbers of anti-diabetic medication, fasting blood glucose, systolic blood pressure and atrial fibrillation |
|  |  |  |  |  |  |  |  |  |  |  | 200-240 mg/dL vs. <160 mg/dL | HR 1.31 (1.25-1.38) |  |
|  |  |  |  |  |  |  |  |  |  |  | >=240 mg/dL vs. <160 mg/dL | HR 1.68 (1.59-1.77) |  |
|  |  |  |  |  |  |  |  | FPG |  |  | <100 mg/dL vs. 100-140 mg/dL | HR 1.19 (1.13-1.25) | Age, sex, smoking, regular exercise, body mass index, chronic kidney disease, duration of diabetes, numbers of anti-diabetic medication, systolic blood pressure, total cholesterol and atrial fibrillation |
|  |  |  |  |  |  |  |  |  |  |  | 140-160 mg/dL vs. 100-140 mg/dL | HR 1.03 (0.98-1.07) |  |
|  |  |  |  |  |  |  |  |  |  |  | 160-180 mg/dL vs. 100-140 mg/dL | HR 1.12 (1.06-1.18) |  |
|  |  |  |  |  |  |  |  |  |  |  | >=180 mg/dL vs. 100-140 mg/dL | HR 1.34 (1.29-1.40) |  |
| Li 2014 | Retrospective cohort study | UK | Patients with T2DM | 57 ± 8 | 45.6 | 21,998 | 9 | LDL-C | Cardiovascular events (angina pectoris, myocardial infarction (MI), coronary revascularization and unspecified ischemic heart disease) | 621 | 2.6-3.3 mmol/L vs. <2.6 mmol/L | HR 1.43 (1.03-1.99) | HDL-c, Triglycerides, HbA1C, Age at cohort entry, Gender, BMI, Smoking, Alcohol abuse, Hypertension, Peripheral vascular disease, Current diabetes treatment, Diabetes duration, Cardiovascular medications, Baseline lipid-modifying therapy (LMT) |
|  |  |  |  |  |  |  |  |  |  |  | 3.3-4.1 mmol/L vs. <2.6 mmol/L | HR 1.85 (1.32-2.58) |  |
|  |  |  |  |  |  |  |  |  |  |  | >4.1 mmol/L vs. <2.6 mmol/L | HR 2.77 (1.93-3.99) |  |
|  |  |  |  |  |  |  |  | HDL-C |  |  | 1.0-1.55 mmol/L vs. <1.0 mmol/L in female and 1.3-1.55 mmol/L vs. <1.3 mmol/L in male | HR 0.83 (0.69-0.99) |  |
|  |  |  |  |  |  |  |  |  |  |  | >=1.55 mmol/L vs. <1.0 mmol/L in female and >=1.55 mmol/L vs. <1.3 mmol/L in male | HR 0.85 (0.65-1.11) |  |
|  |  |  |  |  |  |  |  | TGs |  |  | 1.7-2.25 mmol/L vs. <1.7 mmol/L | HR 1.05 (0.85-1.29) |  |
|  |  |  |  |  |  |  |  |  |  |  | >2.25 mmol/L vs. <1.7 mmol/L | HR 1.03 (0.85-1.26) |  |
| Liu 2017 | Prospective cohort study | China | Patients with T2DM | 52.8 ± 9.5 | 51.9 | 1,004 | 7.8 | FPG | CHD (acute MI or death due to CHD) | 20 | <70 mg/dl vs. 70-99 mg/dl | HR 1.49 (0.60-3.72) | Age (every 10 years), gender, body mass index, smoking status (never smoker, former smoker, current smoker), drinking status (never drinker, drinker), work-related physical activity, education, hypertension status (yes vs no), high triglyceride (yes vs no), region (south vs north), area (urban vs rural), family history of atherosclerotic cardiovascular disease (ASCVD) |
|  |  |  |  |  |  |  |  |  |  |  | 100-125 mg/dl vs. 70-99 mg/dl | HR 1.18 (0.77-1.83) |  |
|  |  |  |  |  |  |  |  |  |  |  | >=126 mg/dl vs. 70-99 mg/dl | HR 2.17 (1.29-3.63) |  |
| Moosaie 2020 | Case-cohort study | Iran | Patients with T2DM | 56.8 ± 9.89 | 52.9 | 1,057 | 5 (median) | Lp(a) | CVD (non-fatal coronary artery disease, non-fatal coronary artery disease) | 242 | NA | OR 1.007 (1.001-1.013) | Age, sex, systolic blood pressure, HbA1c, BMI, use of anti-dyslipidemic drug, eGFR, triglycerides, LDL, HDL, non-HDL cholesterol, and waist/hip ratio |
|  |  |  |  |  |  |  |  |  |  |  | ≥calculated cutoff | OR 2.226 (1.573-3.148) |  |
|  |  |  |  |  |  |  |  | Apo B |  |  | NA | OR 1.006 (1.001-1.016) |  |
|  |  |  |  |  |  |  |  |  |  |  | ≥calculated cutoff | OR 1.866 (0.529-0.968) |  |
|  |  |  |  |  |  |  |  | Apo A1 |  |  | NA | OR 0.975 (0.962-1.003) |  |
|  |  |  |  |  |  |  |  |  |  |  | ≥calculated cutoff | OR 1.410 (0.960-2.070) |  |
| Paul Chubb 2016 | Cohort study | Western Australia | Patients with T2DM | 64.1 ± 11.3 | 51.3 | 905 | 12 (mean) | Bicarbonate | Coronary heart disease (first ever hospitalization for/with CHD or death due to cardiac causes or sudden death) | 361 | Low quintile vs. highest quintile (Q5) | HR 1.39 (0.97-1.98) Q1 1.23 (0.88-1.72) Q2 0.97 (0.71-1.31) Q3 0.92 (0.64-1.32) Q4 | Age as time scale, age at baseline, sex, not fluent in English, diabetes duration, loge(serum triglycerides), loge(urinary albumin:creatinine ratio), peripheral sensory neuropathy, peripheral arterial disease |
|  |  |  |  |  |  |  |  |  |  |  | Each 1 mmol/L increase | HR 0.95 (0.92–0.99) |  |
| Qi 2012 | Cohort study | America | Patients with T2DM | 59.3 ± 7.99 | 56.93 | 2,338 | 12; 16 | Lp(a) | CHD (fatal or non-fatal myocardial infarction (MI) or coronary artery bypass grafting) | 589 | Per 1-SD higher log-transformed Lp(a) levels (The SD of log-transformed Lp(a) was 1.4 in both men and women) | RR 1.05 (0.95-1.16) | Age, fasting status, smoking, alcohol intake, physical activity, duration of diabetes, insulin use, aspirin use, cholesterol-lowering medication use, family history of MI, history of hypertension, BMI, LDL cholesterol, HDL cholesterol, triglycerides, A1C, and hormone replacement therapy use (women only) |
| Shai 2005 | Cohort study | US | Patients with T2DM | 30-55 | 100 | 921 | 7.4 (mean) | Lp(a) | CHD (fatal CHD, non-fatal MI and CABG/PTCA) | 122 | 1.01-6.3 umol/L (the extreme quintiles) vs. 0-0.09 umol/L | RR 1.95 (1.07-3.56) | Age (<49, 50–54, 55–59, 60–64, 65+ years), smoking (current, past, never), BMI (<23, 23–25, 25–28, 28–30, 30–34, 35+ kg/m2), alcohol intake (0, 0.1–4.9, 5+ g/day), physical activity (0–0.9, 1–1.9, 2–3.9, 4+ h/week), postmenopausal hormone use (premenopausal, current, past, never, missing), aspirin use (non-daily, daily), parental history of MI, history of hypertension and levels of HbA1c (quintiles), TGs, LDL-c and HDL-c |
| Sone 2005 | Prospective study | Japan | Patients with T2DM | 58.4 ± 7.4 | 45.9 | 1,424 | 8 | TGs | CHD (myocardial infarction, angina pectoris) | 62 | ≥150 mg/dL vs. <150 mg/dL | HR 2.9 (1.6-5.3) M 1.7 (0.6-4.4) F | NA |
|  |  |  |  |  |  |  |  | HDL-C |  |  | ≤40 mg/dL vs. >40 mg.dL | HR 1.8 (0.9-3.5) M 1.5 (0.6-3.6) F |  |
|  |  |  |  |  |  |  |  | LDL-C |  |  | ≥120 mg/dL vs. <120 mg.dL | HR 2.1 (1.1-3.9) M 1.2 (0.5-3.2) F |  |
| Sone 2011 | Prospective study | Japan | Patients with T2DM | 58.2 | 46.9 | 1,771 | 7.86 (median) | LDL-C | CHD (angina pectoris and myocardial infarction) | 109 | Per 1 mmol/L | HR 1.61 (1.30-1.98) | Gender, age, diabetes duration, body mass index, systolic blood pressure, HbA1c, HDL cholesterol, triglycerides, smoking status, and alcohol intake |
|  |  |  |  |  |  |  |  |  |  |  | Per 1 SD | HR 1.49 (1.25-1.77) |  |
|  |  |  |  |  |  |  |  | HDL-C |  |  | Per 1 mmol/L | HR 0.99 (0.56-1.74) | Gender, age, diabetes duration, body mass index, systolic blood pressure, HbA1c, LDL cholesterol, triglycerides, smoking status, and alcohol intake |
|  |  |  |  |  |  |  |  |  |  |  | Per 1 SD | HR 1.00 (0.78-1.27) |  |
|  |  |  |  |  |  |  |  | TGs |  |  | Per 1 SD (log-transformed) | HR 1.54 (1.22-1.94) | Gender, age, diabetes duration, body mass index, systolic blood pressure, HbA1c, LDL cholesterol, HDL cholesterol, smoking status, and alcohol intake |
|  |  |  |  |  |  |  |  | Lp(a) |  |  | Per 1 SD (log-transformed) | HR 1.15 (0.93-1.43) | Gender, age, diabetes duration, body mass index, systolic blood pressure, HbA1c, LDL cholesterol, HDL cholesterol, triglycerides, smoking status, and alcohol intake |
|  |  |  |  |  |  |  |  | FPG |  |  | Per 1 mmol/L | HR 0.99 (0.91-1.09) |  |
|  |  |  |  |  |  |  |  |  |  |  | Per 1 SD | HR 0.99 (0.79-1.23) |  |
| Stacey 2019 | Cohort study | US | Patients with T2DM | 72 ± 5.3 | 51 | 608 | 6 | FPG | Unrecognized MI | 459 | 100-125 mg/dL vs. <100 mg.dL | HR 1.01 (0.82-1.24) | Age, race, gender, body mass index, hypertension, anti-hypertensive medication use, total cholesterol, HDL cholesterol, lipid-lowering medication use, smoking status |
|  |  |  |  |  |  |  |  |  |  |  | >125 mg/dL vs. <100 mg.dL | HR 1.37 (1.02-1.81) |  |
|  |  |  |  |  |  |  |  | 2 Hour Glucose |  |  | <139 mg/dL vs. 140-199 mg/dL | HR 1.03 (0.83-1.30) |  |
|  |  |  |  |  |  |  |  |  |  |  | >200 mg/dL vs. 140-199 mg/dL | HR 1.03 (0.82-1.29) |  |
| Tanaka 2013 | Secondary analysis | Japan | Patients with T2DM | 62.1 ± 8.6 | 49.9 | 1,748 | 7.2 (median) | Non-HDL-C | Fatal or nonfatal CHD (angina pectoris or myocardial infarction) | 96 | Per 1 mmol/L higher | HR 1.56 (1.26-1.93) | NA |
| Turner 1998 | Prospective study | UK | Patients with T2DM | 52 ± 9 | 41.9 | 3,055 | 7.9 (median) | HDL-C | CAD (fatal or non-fatal myocardial infarction or clinical angina, fatal or non-fatal myocardial infarction) | 335 | ≥0.95 to <1.15 mmol/L vs. ＜0.95 mmol/L | HR 0.90 (0.71-1.15) | Age and sex |
|  |  |  |  |  |  |  |  |  |  |  | ≥1.15 mmol/L vs. ＜0.95 mmol/L | HR 0.55 (0.41-0.73) |  |
|  |  |  |  |  |  |  |  | LDL-C |  |  | ≥3.02 to <3.89 mmol/L vs. ＜3.02 mmol/L | HR 1.41 (1.05-1.90) |  |
|  |  |  |  |  |  |  |  |  |  |  | >3.89 mmol/L vs. ＜3.02 mmol/L | HR 2.26 (1.70-3.00) |  |
| Wan 2022 | Prospective study | UK | Patients with T2DM | 59.3 ± 7.12 | 38.9 | 15,103 | 11.2 (median) | 25(OH)D | Ischemic heart disease (angina pectoris, acute myocardial infarction, subsequent ST elevation (STEMI) and non-ST elevation (NSTEMI) myocardial infarction, certain current complications following ST elevation (STEMI) and non-ST elevation (NSTEMI) myocardial infarction (within the 28 day period), other acute ischemic heart diseases, chronic ischemic heart disease) | 2,195 | 25.0-49.9 nmol/L vs. < 25.0 nmol/L | HR 0.85 (0.76-0.95) | Age (continuous), sex (male or female), and ethnicity (white or other), the Townsend Deprivation Index (continuous), total physical activity level (MET-hour/week; continuous), BMI (<25.0, 25.0–29.9, or ≥30.0 kg/m2), education (college or university degree; A/AS levels or equivalent or O levels/GCSEs or equivalent; other professional qualifications; or none of the above), healthy diet (yes or no), smoking status (never, former, or current smokers), drinking status (never or special occasions only; 1–3 times/month or 1–2 times/week; 3–4 times/week; or daily or almost daily), season of blood draw (March to May, June to August, September to November, or December to February), time spent outdoors in summer (hours/day; continuous), eGFR (≤90 or >90 mL/min/1.73 m2), history of hypertension (yes or no), and hypercholesterolemia (yes or no), diabetes (≤3, 3–10, or >10 years), diabetic medication use (no insulin or pills, only diabetes pills, or insulin and/or others), and HbA1c (<7.0% or ≥7.0%) |
|  |  |  |  |  |  |  |  |  |  |  | 50.0-74.9 nmol/L vs. < 25.0 nmol/L | HR 0.73 (0.64-0.84) |  |
|  |  |  |  |  |  |  |  |  |  |  | ≥ 75.0 nmol/L vs. < 25.0 nmol/L | HR 0.69 (0.56-0.84) |  |
|  |  |  |  |  |  |  |  |  |  |  | Natural log-transformed 25(OH)D | HR 0.77 (0.70-0.85) |  |
| Wang 2017 | Cohort study | China | Patients with T2DM | 64.3 ± 7.5 | 53.4 | 2,918 | 4.4 | Total bilirubin | Coronary heart disease (non-fatal myocardial infarction, stable angina, unstable angina, unspecified CHD, and CHD death) | 440 | High quartile vs. lowest quartile | HR 0.95 (0.72-1.35) Q2 0.89 (0.68-1.17) Q3 0.80 (0.60-1.06) Q4 | Age, sex, central obesity, education level, smoking status, drinking status, physical activity, family history of coronary heart disease，systolic pressure, triglyceride, high-density lipoproteins, use of medications (hypotensor, lipid-lowering, aspirin), and liver function (alkaline phosphatase, alanine aminotransferase, aspartate aminotransferase)，fasting plasma glucose, use of antidiabetic, and duration of diabetes |
|  |  |  |  |  |  |  |  | Direct bilirubin |  |  | High quartile vs. lowest quartile | HR 0.65(0.50-0.86) Q2 0.79(0.60-1.04) Q3 0.77(0.58-1.02) Q4 |  |
|  |  |  |  |  |  |  |  | Indirect bilirubin |  |  | High quartile vs. lowest quartile | HR 0.78 (0.60-1.03) Q2 0.84 (0.64-1.10) Q3 0.75 (0.56-0.99) Q4 |  |
| ***DM→STROKE*** | |  |  |  |  |  |  |  |  |  |  |  |  |
| Abu-Lebdeh 2001 | Cohort | USA | Patients with T2DM | 57 ± 12 | 53 | 449 | 13 (mean) | FPG | Cerebrovascular disease (transient ischemic attacks, ischemic strokes, and stroke deaths) | 115 | Per 1 natural log unit higher | HR 1.69 (1.06-2.70) | NA |
| Colhoun 2011 | Nested case-control study | U.K. and Ireland | Patients with T2DM | 61.7 ± 1.3 | 28.6 | 718 | 3.9 (median) | sRAGE | Stroke | 44 | A doubling of the sRAGE level | HR 0.63 (0.37-1.09) | Age, sex, and treatment group, lipids, BMI, ethnicity, smoking, SBP, diabetes duration, and HbA2c, baseline eGFR and albuminuria |
|  |  |  |  |  |  |  |  | esRAGE |  |  | A doubling of the esRAGE level | HR 0.81 (0.52-1.24) |  |
| Engström 2003 | Cohort study | Sweden | Patients with T2DM | 46.9 | 0 | 321 | 18.7 ± 3.7 | Five ISPs (fibrinogen, orosomucoid, α1-antitrypsin, ceruloplasmin, and haptoglobin) | Stroke (subarachnoid hemorrhage, intracerebral hemorrhage, ischemic stroke, unspecified stroke) | 21 | Two to five ISPs in top quartile vs. Zero to one ISP in top quartile | RR 2.4 (0.98-5.8) | Age and ISP levels |
|  |  |  |  |  |  |  |  |  | Ischemic stroke | 19 | Two to five ISPs in top quartile vs. Zero to one ISP in top quartile | RR 2.0 (0.80-5.0) | Age |
| Gori 2016 | Cohort study | America | Patients with T2DM | 63 | 52 | 1,510 | 13.1 (median) | NT-pro-BNP | Stroke | NA | Per 1 SD higher | HR 1.44 (0.93-2.22) | Age, sex, race, and field center, smoking status, log-transformed pack-years of cigarettes (packs of cigarettes smoked per day times number of years smoked), BMI, waist-to-hip ratio, systolic blood pressure, hypertension medication use, lipid-lowering medication use, aspirin use, education level, total-to-HDL cholesterol ratio, log-transformed triglycerides, and duration of diabetes, conventional measures of diabetes complications (retinopathy, nephropathy, PAD) and ECG abnormalities, cardiac biomarkers (abnormal TnT and abnormal NTproBNP) |
|  |  |  |  |  |  |  |  | TnT |  |  | Per 1 SD higher | HR 2.08 (1.34-3.25) |  |
| Hayashi 2009 | Prospective cohort study | Japan | Patients with T2DM | 67.4 ± 9.5 | 48.23 | 4,014 | 2 | LDL-C | Cerebrovascular disease (stroke) | NA | NA | OR 1.099 (0.98-1.23) <65Yr 1.067 (0.76-1.44) 65-74Yr 1.128 (0.64-1.59) >75Yr | Gender |
|  |  |  |  |  |  |  |  | HDL-C |  |  | NA | OR 0.888 (0.64-1.48) <65Yr 0.758 (0.53-0.98) 65-74Yr 0.511 (0.24-0.92) >75Yr |  |
|  |  |  |  |  |  |  |  | TGs |  |  | NA | OR 1.147 (0.68-2.04) <65Yr 1.070 (0.69-1.67) 65-74Yr 1.355 (0.75-2.56) >75Yr |  |
| Hayashi 2011 | Prospective cohort study | Japan | Patients with T2DM | 70.4 (median) | 49.85 | 4,014 | 2 | LDL-C | Cerebrovascular accident (CVA) (stroke with neurological deficit) | 70 | NA | OR 0.99 (0.96-1.14) <=70Yr, HgbA1C=<7.0 0.98 (0.79-1.07) <=70Yr, 7.0<HgbA1C 0.96 (0.74-1.10) >70Yr, HgbA1C=<7.0 1.04 (0.88-1.21) >70Yr, 7.0<HgbA1C | NA |
|  |  |  |  |  |  |  |  | HDL-C |  |  | NA | OR 0.78 (0.49-1.18) <=70Yr, HgbA1C=<7.0 0.43 (0.23-0.78) <=70Yr, 7.0<HgbA1C 0.61 (0.38-0.93) >70Yr, HgbA1C=<7.0 0.71 (0.49-1.02) >70Yr, 7.0<HgbA1C |  |
|  |  |  |  |  |  |  |  | TGs |  |  | NA | OR 1.00 (0.98-1.02) <=70Yr, HgbA1C=<7.0 1.01 (0.97-1.07) <=70Yr, 7.0<HgbA1C 1.01 (0.91-1.07) >70Yr, HgbA1C=<7.0 1.05 (0.97-1.19) >70Yr, 7.0<HgbA1C |  |
| Hayashi 2013 | Prospective cohort study | Japan | Patients with T2DM | 67.4 ± 9.5 | 48.2 | 4,014 | 5.5 | FPG | Ischemic stroke or primary intracerebral hemorrhage | 104 | Per 10 mg/dL higher | HR 1.005 (0.995-1.005) total 1.003 (0.990-1.018) <65Yr 1.002 (0.995-1.008) 65-74Yr 0.998 (0.986-1.008) >75Yr | NA |
|  |  |  |  |  |  |  |  | LDL-C |  |  | Highest quartile vs. lowest quartile | HR 1.009(0.912-1.191) total 1.005 (1.001-1.100) <65Yr 1.015 (0.892-1.136) 65-74Yr 0.997 (0.982-1.012) >75Yr |  |
|  |  |  |  |  |  |  |  | HDL-C |  |  | Highest quartile vs. lowest quartile | HR 0.742 (0.596-0.901) total 0.715 (0.591-1.191) <65Yr 0.750 (0.494-1.000) 65-74Yr 0.536 (0.320-0.851) >75Yr |  |
|  |  |  |  |  |  |  |  | TGs |  |  | Highest quartile vs. lowest quartile | HR 1.132 (0.908-1.302) total 1.053 (0.658-1.742) <65Yr 1.253 (0.900-1.780) 65-74Yr 1.169 (0.746-1.853) >75Yr |  |
|  |  |  |  |  |  |  |  | Non-HDL-C |  |  | Highest quartile vs. lowest quartile | HR 0.981 (0.945-1.019) total 1.021 (1.003-1.141) <65Yr 0.942 (0.872-1.013) 65-74Yr 1.012 (0.954-1.077) >75Yr |  |
| Li 2014 | Retrospective cohort study | UK | Patients with T2DM | 57 ± 8 | 45.6 | 21,998 | 9 | TGs | Cerebrovascular events (ischemic stroke/transient ischemic attack) | 274 | 1.7-2.25 mmol/L vs.<1.7 mmol/L | HR 1.16 (0.83-1.63) | LDL-c, HDL-c, HbA1C, Age at cohort entry, Gender, BMI, Smoking, Alcohol abuse, Hypertension, Peripheral vascular disease, Current diabetes treatment, Diabetes duration, Cardiovascular medications, Baseline lipid-modifying therapy (LMT) |
|  |  |  |  |  |  |  |  |  |  |  | >2.25 mmol/L vs. <1.7 mmol/L | HR 1.36 (1.01-1.85) |  |
| Liu 2017 | Prospective study | China | Patients with T2DM | 52.8 ± 9.5 | 51.9 | 1,004 | 7.8 | FPG | Stroke | 44 | <70 mg/dl vs. 70-99 mg/dl | HR 1.30 (0.75-2.24) | Age (every 10 years), gender, body mass index, smoking status (never smoker, former smoker, current smoker), drinking status (never drinker, drinker), work-related physical activity, education, hypertension status (yes vs no), high triglyceride (yes vs no), region (south vs north), area (urban vs rural), family history of atherosclerotic cardiovascular disease (ASCVD) |
|  |  |  |  |  |  |  |  |  |  |  | 100-125 mg/dl vs. 70-99 mg/dl | HR 0.96 (0.74-1.25) |  |
|  |  |  |  |  |  |  |  |  |  |  | >=126 mg/dl vs. 70-99 mg/dl | HR 1.51 (1.07-2.13) |  |
| Protopsaltis 2008 | Prospective study | Greece | Patients with T2DM | 60.4 ± 9.6 | 46 | 599 | 10.1 (median) | LDL-C | Ischemic stroke | 78 | Per 1 mg/dL | HR 1.009 (0.992-1.014) | Gender, age, smoking, body mass index, HbA1C, lipids, and diabetes duration |
|  |  |  |  |  |  |  |  | TC |  |  | Per 1 mg/dL | HR 1.005 (0.796-1.019) |  |
|  |  |  |  |  |  |  |  | TGs |  |  | ≥150 mg/dL vs. <150 mg/dL | HR 1.032 (0.900-1.153) |  |
| Shen 2019 | Retrospective cohort study | America | Patients with T2DM | 66.5 ± 0.1 | 23 | 67,544 | 3 (mean) | HDL-C | Stroke (ischemic or hemorrhagic) | 8,496 | 30-39.9 mg/dL vs. <30 mg/dL | HR 0.86 (0.80-0.92) | Age, race, body mass index, systolic blood pressure, HbA1c, low-density lipoprotein cholesterol, triglycerides, estimated GFR, smoking, use of antihypertensive drugs, use of diabetes medications, and use of lipid-lowering agents |
|  |  |  |  |  |  |  |  |  |  |  | 40-49.9 mg/dL vs. <30 mg/dL | HR 0.77 (0.72-0.83) |  |
|  |  |  |  |  |  |  |  |  |  |  | 50-59.9 mg/dL vs. <30 mg/dL | HR 0.71 (0.65-0.77) |  |
|  |  |  |  |  |  |  |  |  |  |  | 60-69.9 mg/dL vs. <30 mg/dL | HR 0.71 (0.64-0.80) |  |
|  |  |  |  |  |  |  |  |  |  |  | 70-79.9 mg/dL vs. <30 mg/dL | HR 0.77 (0.66-0.91) |  |
|  |  |  |  |  |  |  |  |  |  |  | >=80 mg/dL vs. <30 mg/dL | HR 0.69 (0.56-0.85) |  |
| Sone 2005 | Prospective study | Japan | Patients with T2DM | 58.4 ± 7.4 | 45.9 | 1,424 | 8 | LDL-C | Stroke | 59 | ≥120 mg/dL vs. <120 mg/dL | HR 0.9 (0.5-1.8) M 0.6 (0.3-1.3) F | NA |
|  |  |  |  |  |  |  |  | HDL-C |  |  | ≤40 mg/dL vs. >40 mg/dL | HR 1.0 (0.4-2.5) M 1.3 (0.6-2.9) F |  |
|  |  |  |  |  |  |  |  | TGs |  |  | ≥150 mg/dL vs. <150 mg/dL | HR 1.1 (0.5-2.4) M 0.7 (0.2-1.9) F |  |
| Sone 2011 | Prospective study | Japan | Patients with T2DM | 58.2 | 46.9 | 1,771 | 7.86 (median) | Lp(a) | Stroke | 85 | Per 1 SD higher (log-transformed) | HR 1.17 (0.92-1.49) | Gender, age, diabetes duration, body mass index, systolic blood pressure, HbA1c, LDL cholesterol, HDL cholesterol, triglycerides, smoking status, and alcohol intake |
|  |  |  |  |  |  |  |  | LDL-C |  |  | Per 1 mmol/L higher | HR 1.00 (0.76-1.32) | Gender, age, diabetes duration, body mass index, systolic blood pressure, HbA1c, HDL cholesterol, triglycerides, smoking status, and alcohol intake |
|  |  |  |  |  |  |  |  |  |  |  | Per 1 SD higher | HR 1.00 (0.79-1.26) |  |
|  |  |  |  |  |  |  |  | HDL-C |  |  | Per 1 mmol/L higher | HR 0.86 (0.46-0.90) | Gender, age, diabetes duration, body mass index, systolic blood pressure, HbA1c, LDL cholesterol, triglycerides, smoking status, and alcohol intake |
|  |  |  |  |  |  |  |  |  |  |  | Per 1 SD higher | HR 0.94 (0.72-1.23) |  |
|  |  |  |  |  |  |  |  | TGs |  |  | Per 1 SD higher (log-transformed) | HR 1.13 (0.86-1.46) | Gender, age, diabetes duration, body mass index, systolic blood pressure, HbA1c, LDL cholesterol, HDL cholesterol, smoking status, and alcohol intake |
|  |  |  |  |  |  |  |  | FPG |  |  | Per 1 mmol/L higher | HR 1.02 (0.91-1.13) | Gender, age, diabetes duration, body mass index, systolic blood pressure, HbA1c, LDL cholesterol, HDL cholesterol, triglycerides, smoking status, and alcohol intake |
|  |  |  |  |  |  |  |  |  |  |  | Per 1 SD higher | HR 1.04 (0.80-1.35) |  |
| Tanaka 2013 | Secondary analysis | Japan | Patients with T2DM | 62.1 ± 8.6 | 49.9 | 1,748 | 7.2 (median) | Non-HDL-C | Fatal or nonfatal stroke | 89 | Per 1 mmol/L higher | HR 1.38 (1.10-1.74) | NA |
| Wan 2022 | Prospective study | UK | Patients with T2DM | 59.3 ± 7.12 | 38.9 | 15,103 | 11.2 (median) | 25(OH)D | Stroke | 702 | 25.0-49.9 nmol/L vs. < 25.0 nmol/L | HR 0.93 (0.76-1.13) | Age (continuous), sex (male or female), and ethnicity (white or other), the Townsend Deprivation Index (continuous), total physical activity level (MET-hour/week; continuous), BMI (<25.0,25.0–29.9, or≥30.0 kg/m2), education (college or university degree; A/AS levels or equivalent or O levels/GCSEs or equivalent; other professional qualifications; or none of the above), healthy diet (yes or no), smoking status (never, former, or current smokers), drinking status (never or special occasions only; 1–3 times/month or 1–2 times/week; 3–4 times/week; or daily or almost daily), season of blood draw (March to May, June to August, September to November, or December to February), time spent outdoors in summer (hours/day; continuous), eGFR (≤90 or >90 mL/min/1.73 m2), history of hypertension (yes or no), and hypercholesterolemia (yes or no), diabetes (≤3, 3–10, or >10 years), diabetic medication use (no insulin or pills, only diabetes pills, or insulin and/or others), and HbA1c (<7.0% or ≥7.0%) |
|  |  |  |  |  |  |  |  |  |  |  | 50.0-74.9 nmol/L vs. < 25.0 nmol/L | HR 0.92 (0.73-1.16) |  |
|  |  |  |  |  |  |  |  |  |  |  | ≥ 75.0 nmol/L vs. < 25.0 nmol/L | HR 0.74 (0.52-1.06) |  |
|  |  |  |  |  |  |  |  |  |  |  | Natural log-transformed 25(OH)D | HR 0.92 (0.78-1.09) |  |
| ***DM→CHD or STROKE*** | |  |  |  |  |  |  |  |  |  |  |  |  |
| Aroner 2016 | Case-cohort study | America | Patients with T2DM | 62.32 ± 10.01 | 51 | 2,647 | 6.0 (median) | Fetuin-A | Cardiovascular disease events (myocardial infarction (MI), resuscitated cardiac arrest, and stroke) | 31 | 0.43-0.51 g/L vs. 0.20-0.43 g/L | HR 3.19 (1.00-10.14) | Age, sex, racial/ethnic group, field center, smoking status, alcohol intake, body mass index, systolic blood pressure, low-density lipoprotein cholesterol, high-density lipoprotein |
|  |  |  |  |  |  |  |  |  |  |  | 0.51-0.94 g/L vs. 0.20-0.43 g/L | HR 1.90 (0.63-5.76) |  |
| Birukov 2020 | Case-cohort study | Germany | Patients with T2DM | 54.3 | 43.7 | 545 | 12.5 (5.0) median (IQR) | NT-proBNP | Macrovascular complications (myocardial infarction or stroke) | 50 | Per doubling | HR 1.37 (1.03-1.83) | Age at diabetes diagnosis (underlying time scale), duration between recruitment and diabetes diagnosis, sex, education, alcohol intake, smoking, physical activity, body mass index, blood pressure, antihypertensive treatment and history of hyperlipidemia,triglycerides, total and high-density lipoprotein cholesterol, estimated glomerular filtration rate, high-sensitivity C-reactive protein, and sex hormones(testosterone, estradiol and sex hormone binding globulin) ,hemoglobin A1c and adiponectin concentrations |
| Birukov 2022 | Cohort study | Germany | Patients with T2DM | 55.0 ± 12.0 | 44 | 587 | 13.4 (10.9-15.6) median (IQR) | Fetuin-A | Macrovascular complications (myocardial infarction, stroke (ischemic,hemorrhagic and undetermined stroke)) | 56 | Per 1 SD (0.06 g/L) | HR 0.95 (0.68-1.34) | Age at diabetes diagnosis (underlying time scale), duration between recruitment and diabetes diagnosis, sex, education, alcohol intake, physical activity, BMI, waist circumference, history of hypertension, hyperlipidemia, antihypertensive and lipid lowering medications at the time of recruitment |
| Hayashi 2008 | Prospective longitudinal cohort study | Japan | Patients with T2DM | 67.4 ± 9.5 | 48.23 | 4,014 | 1 | HDL-C | Incidence of cardiovascular or CVD (fatal and non-fatal myocardial infarction, and other non-fatal events including unstable angina pectoris, angioplasty, stenting, or coronary artery bypass grafting and stroke) | 60 | <1.05 mmol/L vs >1.58 mmol/L | OR 3.97 (1.91-8.20) | Age |
| Howard 2000 | Case-cohort study | America | Patients with T2DM | 45-74 | 65 | 2,034 | 4.8 (mean) | LDL-C | Fatal and nonfatal CVD (CHD and stroke) | 31.8 per 1000 person-years (men) 17.9 per 1000 person-years (women) | Mean of the upper quartile group vs mean of the lowest quartile group 153 vs 71 mg/dL in women,  149 vs 68 mg/dL in men | HR 2.34 (1.53-3.86) F 2.86 (1.74-4.71) M | Age and center (Arizona center, /Oklahoma center/ Dakota center) |
|  |  |  |  |  |  |  |  | HDL-C |  |  | Mean of the upper quartile group vs mean of the lowest quartile group 60 vs 33 mg/dL in women, 58 vs 28 mg/dL in men | HR 0.71 (0.43-1.16) F 0.66 (0.37-1.17) M |  |
|  |  |  |  |  |  |  |  | TGs |  |  | Mean of the upper quartile group vs mean of the lowest quartile group 333 vs 78 mg/dL in women,  391 vs 68 mg/dL in men | HR 1.58 (1.28-1.95) F 1.06 (0.89-1.26) M |  |
| Lee 2020 | Cohort study | Korea | Patients with T2DM | 72.5 | 52 | 227,938 | 5.6 ± 1.2 | FPG | CVD (stroke or myocardial infarction) | 30,843 | <=79 mg/dL vs. 110-124 mg/dL | HR 1.27 (1.10-1.48) | Age at baseline, sex, family income, residential area, smoking status, diabetes duration (>= 5 years/< 5 year), alcohol intake, regular exercise, body mass index, systolic blood pressure, Charlson comorbidity index and total cholesterol |
|  |  |  |  |  |  |  |  |  |  |  | 80-94 mg/dL vs. 110-124 mg/dL | HR 1.14 (1.06-1.22) |  |
|  |  |  |  |  |  |  |  |  |  |  | 95-109 mg/dL vs. 110-124 mg/dL | HR 0.99 (0.94-1.05) |  |
|  |  |  |  |  |  |  |  |  |  |  | 125-139 mg/dL vs. 110-124 mg/dL | HR 1.05 (1.01-1.10) |  |
|  |  |  |  |  |  |  |  |  |  |  | 140-154 mg/dL vs. 110-124 mg/dL | HR 1.12 (1.06-1.18) |  |
|  |  |  |  |  |  |  |  |  |  |  | 155-169 mg/dL vs. 110-124 mg/dL | HR 1.25 (1.17-1.32) |  |
|  |  |  |  |  |  |  |  |  |  |  | 170-184 mg/dL vs. 110-124 mg/dL | HR 1.36 (1.27-1.46) |  |
|  |  |  |  |  |  |  |  |  |  |  | 185-199 mg/dL vs. 110-124 mg/dL | HR 1.47 (1.35-1.60) |  |
|  |  |  |  |  |  |  |  |  |  |  | >=200 mg/dL vs. 110-124 mg/dL | HR 1.76 (1.64-1.88) |  |
| Liu 2017 | Prospective cohort study | China | Patients with T2DM | 52.8 ± 9.5 | 51.9 | 1,004 | 7.8 | FPG | ASCVD (the first occurrence of non-fatal myocardial infarction (MI), CHD death, or fatal or non-fatal stroke) | 64 | <70 mg/dl vs. 70-99 mg/dl | HR 1.35 (0.84-2.15) | Age (every 10 years), gender, body mass index, smoking status (never smoker, former smoker, current smoker), drinking status (never drinker, drinker), work-related physical activity, education, hypertension status (yes vs no), high triglyceride (yes vs no), region (south vs north), area (urban vs rural), and family history of atherosclerotic cardiovascular disease (ASCVD) |
|  |  |  |  |  |  |  |  |  |  |  | 100-125 mg/dl vs. 70-99 mg/dl | HR 1.02 (0.81-1.27) |  |
|  |  |  |  |  |  |  |  |  |  |  | >=126 mg/dl vs. 70-99 mg/dl | HR 1.68 (1.26-2.23) |  |
| Looker 2015 | Nested case–control study | Europe | Patients with T2DM | 68.6 | 42.99 | 2,310 | 6.5 (3.9, 7.9) | ApoCIII | CVD (stroke or acute CHD) | 1,123 | Per 1 SD higher | OR 0.79 (0.71-0.87) | Age, sex, smoking, systolic and diastolic blood pressure, LDL-cholesterol (LDL-C), HDL-cholesterol (HDL-C), triacylglycerol, diabetes duration, HbA1C, BMI, height, estimated GFR (eGFR) calculated using the Modification of Diet in Renal Disease 4-variable (MDRD4) equation, cohort, and current medication (including antihypertensive agents, aspirin, lipid-lowering agents and insulin) |
|  |  |  |  |  |  |  |  | Brain-derived neurotrophic factor |  |  | Per 1 SD higher | OR 0.92 (0.84-1.01) |  |
|  |  |  |  |  |  |  |  | Cathepsin S |  |  | Per 1 SD higher | OR 1.07 (0.97-1.20) |  |
|  |  |  |  |  |  |  |  | Cystatin C |  |  | Per 1 SD higher | OR 1.35 (1.18-1.56) |  |
|  |  |  |  |  |  |  |  | Eotaxin-1 |  |  | Per 1 SD higher | OR 1.02 (0.93-1.13) |  |
|  |  |  |  |  |  |  |  | Factor VII |  |  | Per 1 SD higher | OR 1.02 (0.93-1.13) |  |
|  |  |  |  |  |  |  |  | Fetuin A |  |  | Per 1 SD higher | OR 1.01 (0.92-1.12) |  |
|  |  |  |  |  |  |  |  | hsTnT |  |  | Per 1 SD higher | OR 1.57 (1.40-1.78) |  |
|  |  |  |  |  |  |  |  | Intercellular adhesion molecule 1 |  |  | Per 1 SD higher | OR 1.11 (1.01-1.21) |  |
|  |  |  |  |  |  |  |  | IL-1α |  |  | Per 1 SD higher | OR 1.03 (0.94-1.14) |  |
|  |  |  |  |  |  |  |  | IL-1 receptor antagonist |  |  | Per 1 SD higher | OR 1.13 (1.02-1.25) |  |
|  |  |  |  |  |  |  |  | IL-6 |  |  | Per 1 SD higher | OR 1.33 (1.20-1.47) |  |
|  |  |  |  |  |  |  |  | IL-8 |  |  | Per 1 SD higher | OR 1.03 (0.94-1.13) |  |
|  |  |  |  |  |  |  |  | IL-10 |  |  | Per 1 SD higher | OR 1.05 (0.95-1.16) |  |
|  |  |  |  |  |  |  |  | IL-15 |  |  | Per 1 SD higher | OR 1.18 (1.08-1.30) |  |
|  |  |  |  |  |  |  |  | IL-17 |  |  | Per 1 SD higher | OR 1.03 (0.94-1.12) |  |
|  |  |  |  |  |  |  |  | IL-18 |  |  | Per 1 SD higher | OR 1.02 (0.93-1.12) |  |
|  |  |  |  |  |  |  |  | IL-23 |  |  | Per 1 SD higher | OR 1.12 (1.02-1.22) |  |
|  |  |  |  |  |  |  |  | Macrophage inflammatory protein-1α |  |  | Per 1 SD higher | OR 1.15 (1.04-1.27) |  |
|  |  |  |  |  |  |  |  | Macrophage inflammatory protein-1β |  |  | Per 1 SD higher | OR 1.03 (0.95-1.13) |  |
|  |  |  |  |  |  |  |  | Matrix metalloproteinase-2 |  |  | Per 1 SD higher | OR 1.25 (1.06-1.48) |  |
|  |  |  |  |  |  |  |  | Matrix metalloproteinase-3 |  |  | Per 1 SD higher | OR 1.08 (0.97-1.20) |  |
|  |  |  |  |  |  |  |  | Matrix metalloproteinase-9 |  |  | Per 1 SD higher | OR 1.11 (1.00-1.24) |  |
|  |  |  |  |  |  |  |  | Monocyte chemotactic protein 1 |  |  | Per 1 SD higher | OR 0.97 (0.89-1.06) |  |
|  |  |  |  |  |  |  |  | NT-proBNP |  |  | Per 1 SD higher | OR 1.89 (1.67-2.16) |  |
|  |  |  |  |  |  |  |  | Osteopontin |  |  | Per 1 SD higher | OR 1.04 (0.93-1.16) |  |
|  |  |  |  |  |  |  |  | Osteoprotegerin |  |  | Per 1 SD higher | OR 1.15 (1.04-1.27) |  |
|  |  |  |  |  |  |  |  | sRAGE |  |  | Per 1 SD higher | OR 0.94 (0.85-1.03) |  |
|  |  |  |  |  |  |  |  | Stem cell factor |  |  | Per 1 SD higher | OR 1.05 (0.94-1.18) |  |
|  |  |  |  |  |  |  |  | TNF-α |  |  | Per 1 SD higher | OR 1.06 (0.96-1.16) |  |
|  |  |  |  |  |  |  |  | Vascular endothelial growth factor |  |  | Per 1 SD higher | OR 1.13 (1.03-1.25) |  |
|  |  |  |  |  |  |  |  | Interferon-γ |  |  | (Below median) per 1 SD higher | OR 0.68 (0.38-1.20) |  |
|  |  |  |  |  |  |  |  |  |  |  | (Above median) per 1 SD higher | OR 1.66 (0.84-3.39) |  |
|  |  |  |  |  |  |  |  | IL-3 |  |  | (Below median) per 1 SD higher | OR 0.98 (0.64-1.51) |  |
|  |  |  |  |  |  |  |  |  |  |  | (Above median) per 1 SD higher | OR 0.77 (0.50-1.20) |  |
|  |  |  |  |  |  |  |  | IL-4 |  |  | (Below median) per 1 SD higher | OR 1.07 (0.72-1.60) |  |
|  |  |  |  |  |  |  |  |  |  |  | (Above median) per 1 SD higher | OR 1.22 (0.76-1.97) |  |
|  |  |  |  |  |  |  |  | IL-7 |  |  | (Below median) per 1 SD higher | OR 1.34 (0.99-1.82) |  |
|  |  |  |  |  |  |  |  |  |  |  | (Above median) per 1 SD higher | OR 1.09 (0.80-1.48) |  |
|  |  |  |  |  |  |  |  | TNF-β |  |  | (Below median) per 1 SD higher | OR 0.59 (0.35-0.97) |  |
|  |  |  |  |  |  |  |  |  |  |  | (Above median) per 1 SD higher | OR 1.41 (0.79-2.58) |  |
| Qi 2012 | Cohort study | America | Patients with T2DM | 59.3 ± 7.99 | 56.93 | 2,338 | 12; 16 | Lp(a) | CVD (fatal or non-fatal myocardial infarction (MI) or coronary artery bypass grafting) or stroke | 677 | Per 1-SD higher log-transformed Lp(a) levels（The SD of log-transformed Lp(a) was 1.4 in both men and women） | RR 1.05 (0.96-1.15) | Age, fasting status, smoking, alcohol intake, physical activity, duration of diabetes, insulin use, aspirin use, cholesterol-lowering medication use, family history of MI, history of hypertension, BMI, LDL cholesterol, HDL cholesterol, triglycerides, A1C, and hormone replacement therapy use (women only) |
| Saeed 2019 | Prospective cohort study | America | Patients with T2DM | 63.1 ± 5.60 | 57 | 1,543 | 13.4 ± 4.75 | Lp(a) | Incident ASCVD (defined as a composite of incident coronary heart disease and ischemic stroke events) | 469 | Per natural-log unit of Lp(a) increase | HR 1.10 (1.01-1.19) Caucasians 1.17 (0.97-1.40) African Americans | Age, gender, total cholesterol, HDL-C, SBP, antihypertensive medication use, current smoking and eGFR |
|  |  |  |  |  |  |  |  |  |  |  | 10-20 mg/dL vs. <10 mg/dL | HR 0.94 (0.66-1.34) Caucasians 0.87 (0.43-1.72) African Americans |  |
|  |  |  |  |  |  |  |  |  |  |  | 20-30 mg/dL vs. <10 mf/dL | HR 0.90 (0.51-1.58) Caucasians 1.02 (0.53-1.94) African Americans |  |
|  |  |  |  |  |  |  |  |  |  |  | 30-<50 mg/dL vs. <10 mg/dL | HR 1.21 (0.81-1.80) Caucasians 1.41 (0.79-2.53) African Americans |  |
|  |  |  |  |  |  |  |  |  |  |  | >=50 mg/dL vs. <10 mg/dL | HR 1.59 (1.18-2.15) Caucasians 1.24 (0.70-2.20) African Americans |  |
| Sluik 2012 | Cohort study | Germany and Netherlands | Patients with T2DM | 57.80 ± 6.68 | NA | 1,280 | 8.2 (median) | GGT | Non-fatal CVD (defined as incident myocardial infarction (MI), ischaemic heart disease or stroke) | 108 | Sex-specific quartile 2 vs. quartile 1 | HR 0.89 (0.45-1.75) | Sex, diabetes duration, insulin use, use of oral blood glucose lowering medication and HbA1c and waist–height ratio, body mass index, self-reported hyperlipidaemia, hypertension, smoking status, alcohol consumption, fruit, vegetable and legume intake, physical activity and educational level and systolic blood pressure, total cholesterol, HDL-cholesterol, triglycerides, C-reactive protein and uric acid |
|  |  |  |  |  |  |  |  |  |  |  | Sex-specific quartile 3 vs. quartile 1 | HR 1.12 (0.58-2.18) |  |
|  |  |  |  |  |  |  |  |  |  |  | Sex-specific quartile 4 vs. quartile 1 | HR 0.83 (0.39-1.79) |  |
|  |  |  |  |  |  |  |  |  | Fatal and non-fatal CVD (defined as incident myocardial infarction (MI), ischaemic heart disease or stroke) | 119 | Sex-specific quartile 2 vs. quartile 1 | HR 0.88 (0.46-1.70) |  |
|  |  |  |  |  |  |  |  |  |  |  | Sex-specific quartile 3 vs. quartile 1 | HR 1.18 (0.62-2.24) |  |
|  |  |  |  |  |  |  |  |  |  |  | Sex-specific quartile 4 vs. quartile 1 | HR 1.21 (0.61-2.42) |  |
| Wu 2021 | Prospective community-based study | China | Patients with T2DM | 55.8 ± 10.6 | 17.3 | 8,244 | 10.4 | HDL-C | CVDs (myocardial infarction, ischemic stroke, or hemorrhagic stroke) | NA | <1.04 mmol/L vs. 1.30-1.42 mmol/L | HR 0.94 (0.64-1.63) | Age; sex (men or women); family history of myocardial infarction, stroke, DM, or hypertension (yes or no); smoking status (never, former, occasionally, or daily); BMI (quartiles); waist circumference (quartiles); LDL cholesterol (quartiles); triglyceride (mmol/L); high-sensitivity C-reactive protein (quartiles); systolic blood pressure (quartiles); diastolic blood pressure (quartiles); glucose (quartiles); estimated glomerular filtration rate (quartiles); alcohol consumption (never, former, occasionally, or daily); education level (primary, middle/high school, or college/university); occupation (white collar, coal miner, or blue collar); physical activity (inactive, moderately active, or vigorously active); monthly salary (<600, 600–800, 800–1000, or>=1000¥); and baseline blood pressure status (normal, prehypertension, or hypertension). The models for participants with DM were in addition adjusted for glucose-lowering drugs |
|  |  |  |  |  |  |  |  |  |  |  | 1.04-1.29 mmol/L vs. 1.30-1.42 mmol/L | HR 1.08 (0.87-1.34) |  |
|  |  |  |  |  |  |  |  |  |  |  | 1.43-1.54 mmol/L vs. 1.30-1.42 mmol/L | HR 1.24 (0.99-1.56) |  |
|  |  |  |  |  |  |  |  |  |  |  | 1.55-1.80 mmol/L vs. 1.30-1.42 mmol/L | HR 1.32 (1.06-1.64) |  |
|  |  |  |  |  |  |  |  |  |  |  | 1.81-2.07 mmol/L vs. 1.30-1.42 mmol/L | HR 1.75 (1.34-2.28) |  |
|  |  |  |  |  |  |  |  |  |  |  | >2.07 mmol/L vs. 1.30-1.42 mmol/L | HR 1.62 (1.19-2.20) |  |
| ***CHD→DM*** | |  |  |  |  |  |  |  |  |  |  |  |  |
| Knudsen 2009 | Prospective cohort study | Norway | STEMI (ST-elevation myocardial infarction) patients treated with primary PCI | 58 (51, 67) | 17.4 | 224 | 0.25 | FPG | Abnormal Glucose Regulation (impaired glucose tolerance (IGT)+impaired glucose tolerance (IGT)+DM) | 50 | ≥ 6.1mmol/L vs. ＜6.1mmol/L | OR 2.62 (1.25, 5.50) | Age, uric acid and gender |
|  |  |  |  |  |  |  |  | Admission plasma glucose |  |  | ≥ 7.7 mmol/L vs. ＜7.7 mmol/L | OR 2.12 (1.03-4.33) | Age |
| Pertiwi 2020 | Cohort study | Dutch | Patients with post-MI | 68.9 ± 5.5 | 19.5 | 3,257 | 3.39 (3.07, 3.46) | Linoleic acid | T2DM | 171 | Per 5% increase | HR 0.73 (0.62-0.86) | Age (continuous; years), sex (men/women), and Alpha Omega Trial treatment code (four categories), physical activity (three categories), smoking status (three categories), educational level (four categories), BMI (continuous; kg/m2), family history of type 2 diabetes (yes/no), total energy intake (excluding calories from alcohol, continuous; kcal/day), alcohol intake (four categories), dietary fiber (g/day; continuous), and dietary cholesterol (mg/day; continuous) |
|  |  |  |  |  |  |  |  |  |  |  | Quintile 2 vs. quintile 1 | HR 0.74 (0.48-1.14) |  |
|  |  |  |  |  |  |  |  |  |  |  | Quintile 3 vs. quintile 1 | HR 0.64 (0.40-1.00) |  |
|  |  |  |  |  |  |  |  |  |  |  | Quintile 4 vs. quintile 2 | HR 0.60 (0.38-0.94) |  |
|  |  |  |  |  |  |  |  |  |  |  | Quintile 5 vs. quintile 2 | HR 0.44 (0.26-0.75) |  |
| Strand 2018 | Cohort study | Norway | Patients with coronary artery disease | 62 (54, 69) | 26.9 | 2,519 | 7.7 (6.4, 8.7) | Trimethyllysi-ne | T2DM | 173 | Per 1 SD increment | OR 1.22 (1.04-1.43) | Age, sex, fasting status, BMI, eGFR, HbA1c, triglyceride and HDL cholesterol levels, and study center |
|  |  |  |  |  |  |  |  | γ-butyrobetaine |  |  |  | OR 0.81 (0.66-0.98) |  |
|  |  |  |  |  |  |  |  | Carnitine |  |  |  | OR 0.93 (0.78-1.10) |  |
|  |  |  |  |  |  |  |  | Acetylcarnitine (C2) |  |  |  | OR 0.99 (0.82-1.20) |  |
|  |  |  |  |  |  |  |  | Propionylcarnitine (C3) |  |  |  | OR 1.05 (0.87-1.27) |  |
|  |  |  |  |  |  |  |  | (Iso)valerylcarnitine (C5) |  |  |  | OR 1.10 (0.92-1.31) |  |
|  |  |  |  |  |  |  |  | Octanoylcarnitine (C8) |  |  |  | OR 1.10 (0.93-1.32) |  |
|  |  |  |  |  |  |  |  | Palmitoylcarnitine (C16) |  |  |  | OR 1.24 (1.04-1.49) |  |
| ***CMM*** | |  |  |  |  |  |  |  |  |  |  |  |  |
| Qiao 2021 | Cohort study | England, Wales, and Scotland (UK biobank) | Free of cardio-metabolic disease | 56.13 ± 8.1 | 55.76 | 353,427 | 8.9  (8.2, 9.6) | HDL-C | CMM (the co-existence of two and more conditions of type 2 diabetes, coronary heart disease (CHD), and stroke) | 3,389 | < 1.3 mmol/L vs. ≥1.3 mmol/L (women) < 1.0 mmol/L vs. ≥1.0 mmol/L (men) | HR 1.50 (1.38-1.62) | Age at baseline and gender, socioeconomic status and ethnicity,smoking status, alcohol drinking and physical activity |
|  |  |  |  |  |  |  |  |  |  |  | Per 1 SD increase | HR 0.79 (0.75-0.83) |  |
|  |  |  |  |  |  |  |  | TGs |  |  | ≥ 1.7 mmol/L vs. ＜1.7 mmol/L | HR 1.42 (1.32-1.53) |  |
|  |  |  |  |  |  |  |  |  |  |  | Per 1 SD increase | HR 1.11 (1.08-1.14) |  |
|  |  |  |  |  |  |  |  | FPG |  |  | ≥ 5.60 mmol/L vs. ＜5.60 mmol/L | HR 2.29 (2.13-2.47) |  |
|  |  |  |  |  |  |  |  |  |  |  | Per 1 SD increase | HR 1.13 (1.12-1.15) |  |
| Singh-Manoux 2018 | Cohort study | UK | Free of cardio-metabolic disease | 50 ± 5 | 32.9 | 8,270 | 23.7 ± 5.9 | TC | CMM | 511 | >=5 mmol/L vs. ＜5 mmol/L | HR 1.32 (0.98-1.77) | Age, sex, ethnicity, marital status, and birth cohort |
|  |  |  |  | NA | NA | 2,501 | 23.7 ± 5.9 |  | First disease to CMM | 511 |  | HR 1.08 (0.80-1.46) |  |
| Tajik 2022 | Prospective cohort study | Finland | Free of cardio-metabolic disease | 52.5 ± 5.2 | 0 | 1,728 | 22.4 (mean) | TGs | CHD + stroke | 146 | Extreme quartile | HR 0.98 (0.59-1.63) | Age, examination year, body mass index, smoking, leisure-time physical activity, education, income, medication, alcohol intake and energy intake |
|  |  |  |  |  |  |  |  |  | CHD + T2DM | 110 |  | HR 1.99 (1.12-3.53) |  |
|  |  |  |  |  |  |  |  |  | Stroke + T2DM | 50 |  | HR 1.66 (0.68-4.04) |  |
|  |  |  |  |  |  |  |  |  | All three | 29 |  | HR 1.18 (0.39-3.57) |  |
|  |  |  |  |  |  |  |  | TC | CHD + stroke | 146 | Extreme quartile | HR 0.96 (0.59-1.56) |  |
|  |  |  |  |  |  |  |  |  | CHD + T2DM | 110 |  | HR 1.35 (0.79-2.31) |  |
|  |  |  |  |  |  |  |  |  | Stroke + T2DM | 50 |  | HR 0.86 (0.36-2.08) |  |
|  |  |  |  |  |  |  |  |  | All three | 29 |  | HR 0.57 (0.17-1.98) |  |
|  |  |  |  |  |  |  |  | VLDL-C | CHD + stroke | 146 | Extreme quartile | HR 1.11 (0.69-1.79) |  |
|  |  |  |  |  |  |  |  |  | CHD + T2DM | 110 |  | HR 1.79 (1.04-3.11) |  |
|  |  |  |  |  |  |  |  |  | Stroke + T2DM | 50 |  | HR 1.65 (0.75-3.65) |  |
|  |  |  |  |  |  |  |  |  | All three | 29 |  | HR 1.93 (0.66-5.06) |  |
|  |  |  |  |  |  |  |  | LDL-C | CHD + stroke | 146 | Extreme quartile | HR 0.98 (0.62-1.57) |  |
|  |  |  |  |  |  |  |  |  | CHD + T2DM | 110 |  | HR 1.01 (0.59-1.72) |  |
|  |  |  |  |  |  |  |  |  | Stroke + T2DM | 50 |  | HR 1.04 (0.47-2.31) |  |
|  |  |  |  |  |  |  |  |  | All three | 29 |  | HR 1.15 (0.14-1.51) |  |
|  |  |  |  |  |  |  |  | HDL-C | CHD + stroke | 146 | Extreme quartile | HR 1.03 (0.64-1.66) |  |
|  |  |  |  |  |  |  |  |  | CHD + T2DM | 110 |  | HR 0.49 (0.40-1.00) |  |
|  |  |  |  |  |  |  |  |  | Stroke + T2DM | 50 |  | HR 0.68 (0.30-1.54) |  |
|  |  |  |  |  |  |  |  |  | All three | 29 |  | HR 0.53 (0.18-1.52) |  |
|  |  |  |  |  |  |  |  | Non-HDL-C | CHD + stroke | 146 | Extreme quartile | HR 0.96 (0.59-1.57) |  |
|  |  |  |  |  |  |  |  |  | CHD + T2DM | 110 |  | HR 1.25 (0.75-2.08) |  |
|  |  |  |  |  |  |  |  |  | Stroke + T2DM | 50 |  | HR 0.69 (0.30-1.58) |  |
|  |  |  |  |  |  |  |  |  | All three | 29 |  | HR 0.45 (0.14-1.53) |  |
|  |  |  |  |  |  |  |  | ApoA1 | CHD + stroke | 146 | Extreme quartile | HR 1.01 (0.61-1.68) |  |
|  |  |  |  |  |  |  |  |  | CHD + T2DM | 110 |  | HR 0.61 (0.35-1.07) |  |
|  |  |  |  |  |  |  |  |  | Stroke + T2DM | 50 |  | HR 1.11 (0.48-2.54) |  |
|  |  |  |  |  |  |  |  |  | All three | 29 |  | HR 0.59 (0.19-1.77) |  |
|  |  |  |  |  |  |  |  | ApoB | CHD + stroke | 146 | Extreme quartile | HR 0.68 (0.30-1.58) |  |
|  |  |  |  |  |  |  |  |  | CHD + T2DM | 110 |  | HR 1.35 (0.78-2.32) |  |
|  |  |  |  |  |  |  |  |  | Stroke + T2DM | 50 |  | HR 1.01 (0.46-2.22) |  |
|  |  |  |  |  |  |  |  |  | All three | 29 |  | HR 0.62 (0.22-1.76) |  |

# Abbreviations

| CMM | Cardiometabolic multimorbidity |
| --- | --- |
| CHD | Coronary heart disease |
| IHD | Ischemic heart disease |
| MI | Myocardial infarction |
| T2DM | Type 2 diabetes mellitus |
| TC | Total cholesterol |
| TGs | Triglycerides |
| HDL-C | High-density lipoprotein (cholesterol) |
| LDL-C | Low-density lipoprotein (cholesterol) |
| NHDL-C | Non-high-density lipoprotein cholesterol |
| Lp(a) | Lipoprotein(a) |
| Apo A1 | Apolipoprotein A1 |
| Apo B | Apolipoprotein B |
| Apo CIII | Apolipoprotein C III |
| FPG | Fasting plasma glucose |
| 2h PG | 2-hour Post-load glucose |
| hs-CRP | Hypersensitive C-reactive protein |
| IL | Interleukin (including IL-1α/1/3/4/6/7/8/10/15/17/18/23) |
| TNF-α | Tumor necrosis factor-α |
| TNF-β | Tumor necrosis factor-β |
| IFN-γ | Interferon-γ |
| NT-proBNP | N-terminal pro-B type natriuretic peptide |
| sRAGE | Soluble receptor of advanced glycation end products |
| esRAGE | Endogenous secretory receptor for advanced glycation end products |
| ISPs | inflammation-sensitive plasma markers |
| ICAM-1 | Intercellular adhesion molecule 1 |
| MMP | Matrix metalloproteinase-2/3/9 |
| OPG | Osteoprotegerin |
| VEGF | Vascular endothelial growth factor |
| GGT | Gamma-glutamyl transferase |
| TnT | Troponin T |
| hs-TnT | High sensitivity Troponin T |
| PRISMA | Preferred Reporting Items for Systematic Reviews and Meta-Analyses |
| NOS | Newcastle‐Ottawa Quality Assessment Scale |
| RevMan | Review Manager |
| OR | Odds ratio |
| RR | Risk ratio |
| HR | Hazard ratio |
| CI | Confidence interval |
